# Recent Developments and Challenges in the Enzymatic Formation of Nitrogen–Nitrogen Bonds

**DOI:** 10.1021/acscatal.4c05268

**Published:** 2024-12-17

**Authors:** Charitomeni Angeli, Sara Atienza-Sanz, Simon Schröder, Annika Hein, Yongxin Li, Alexander Argyrou, Angelina Osipyan, Henrik Terholsen, Sandy Schmidt

**Affiliations:** † Department of Chemical and Pharmaceutical Biology, Groningen Research Institute of Pharmacy, University of Groningen, Antonius Deusinglaan 1, Groningen 9713AV, The Netherlands

**Keywords:** Nitrogen−Nitrogen Bond, NNzymes, Natural Products, Biocatalysis, Heme Enzymes, Cupin Domain

## Abstract

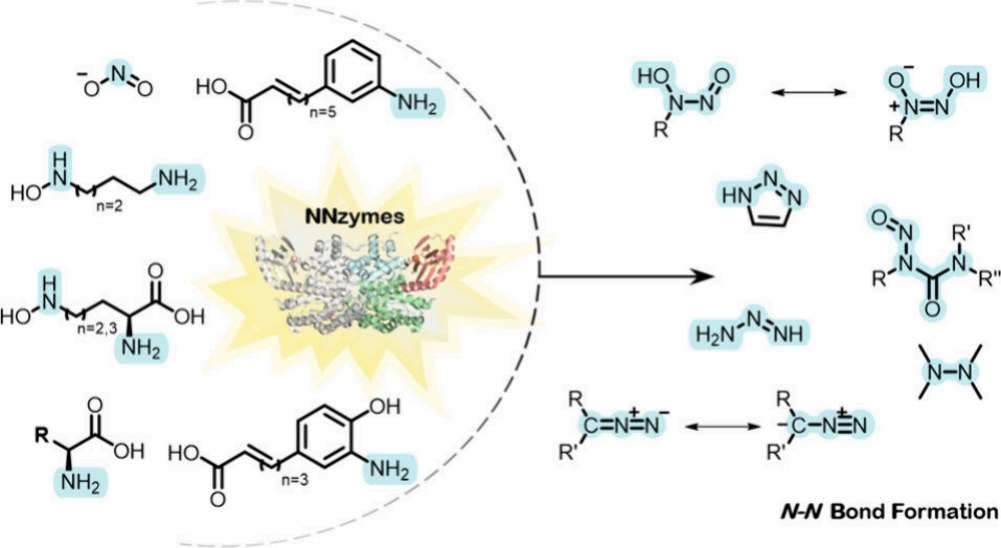

The biological formation of nitrogen–nitrogen (N–N) bonds represents intriguing reactions that have attracted much attention in the past decade. This interest has led to an increasing number of N–N bond-containing natural products (NPs) and related enzymes that catalyze their formation (referred to in this review as NNzymes) being elucidated and studied in greater detail. While more detailed information on the biosynthesis of N–N bond-containing NPs, which has only become available in recent years, provides an unprecedented source of biosynthetic enzymes, their potential for biocatalytic applications has been minimally explored. With this review, we aim not only to provide a comprehensive overview of both characterized NNzymes and hypothetical biocatalysts with putative N–N bond forming activity, but also to highlight the potential of NNzymes from a biocatalytic perspective. We also present and compare conventional synthetic approaches to linear and cyclic hydrazines, hydrazides, diazo- and nitroso-groups, triazenes, and triazoles to allow comparison with enzymatic routes via NNzymes to these N–N bond-containing functional groups. Moreover, the biosynthetic pathways as well as the diversity and reaction mechanisms of NNzymes are presented according to the direct functional groups currently accessible to these enzymes.

## 1 Introduction

**Figure 1:**
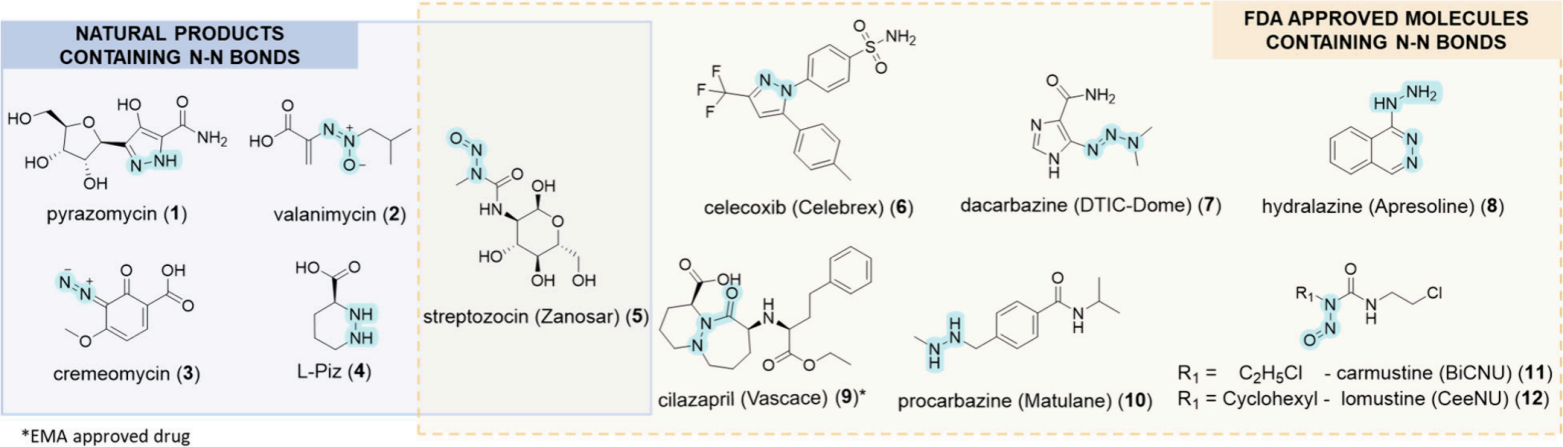
Chemical structures of N–N bond-containing natural products and FDA-approved drugs. Commercial names are mentioned in brackets.

Since the isolation of the first nitrogen–nitrogen (N–N) bond-containing natural product (NP) macrozamin in 1940 from an Australian cycad plant,^1^ hundreds of NPs containing N–N bonds have been reported from a plethora of organisms.^2,3^ These NPs are particularly noteworthy due to their potential as therapeutic agents and precursors for synthesizing biologically active molecules. In addition to NPs, N–N bond containing groups have already been used as key-structural motifs in diverse compounds applied to dyes, agrochemicals, synthetic materials and cosmetics.^3−7^ Currently, the biological activities of N–N bond-containing compounds including pyrazomycin (**1**), valanimycin (**2**), cremeomycin (**3**), derivatives of piperazate (**L-4**) and other related metabolites are being investigated (Figure 1).^3,8−11^ One such compound, the *N*-nitroso-containing NP streptozocin (**5**) is both a NP and an FDA approved drug. Marketed under the brand name Zanosar, it is an antineoplastic agent used to treat pancreatic cancer.^12−15^ Pharmaceutical innovations particularly benefit from synthetic and natural compounds containing N–N bonds, mainly aromatic or nonaromatic heterocycles, because of their diverse range of antiviral, antibacterial, antimalarial and anticancer activities.^3,8,16^ As a matter of fact, each year, the FDA’s CDER (The United States Food and Drug Administration’s Center for Drug Evaluation and Research) approves an increasing number of new drugs containing this intriguing structural motif.^17^ For instance, celecoxib (**6)**, a synthetic pyrazole compound employed for pain relief in arthritis.^18,19^ Dacarbazine (**7**), a chemotherapy drug presents the triazine functionality,^20^ while hydralazine (**8**), which is prescribed for high blood pressure,^21^ contains dual hydrazine groups. The angiotensin converting enzyme (ACE) inhibitor cilazapril (**9**) (EMA approved) includes the common hydrazide functionality.^22^ In addition, the chemotherapy agents procarbazine (**10**), carmustine (**11**), and lomustine (**12**) contain N–N functionalities. The former as a nonderivatized hydrazine group and the latter two include the *N*-nitrosamine functionality (Figure 1).^15,23,24^

**Figure 2:**
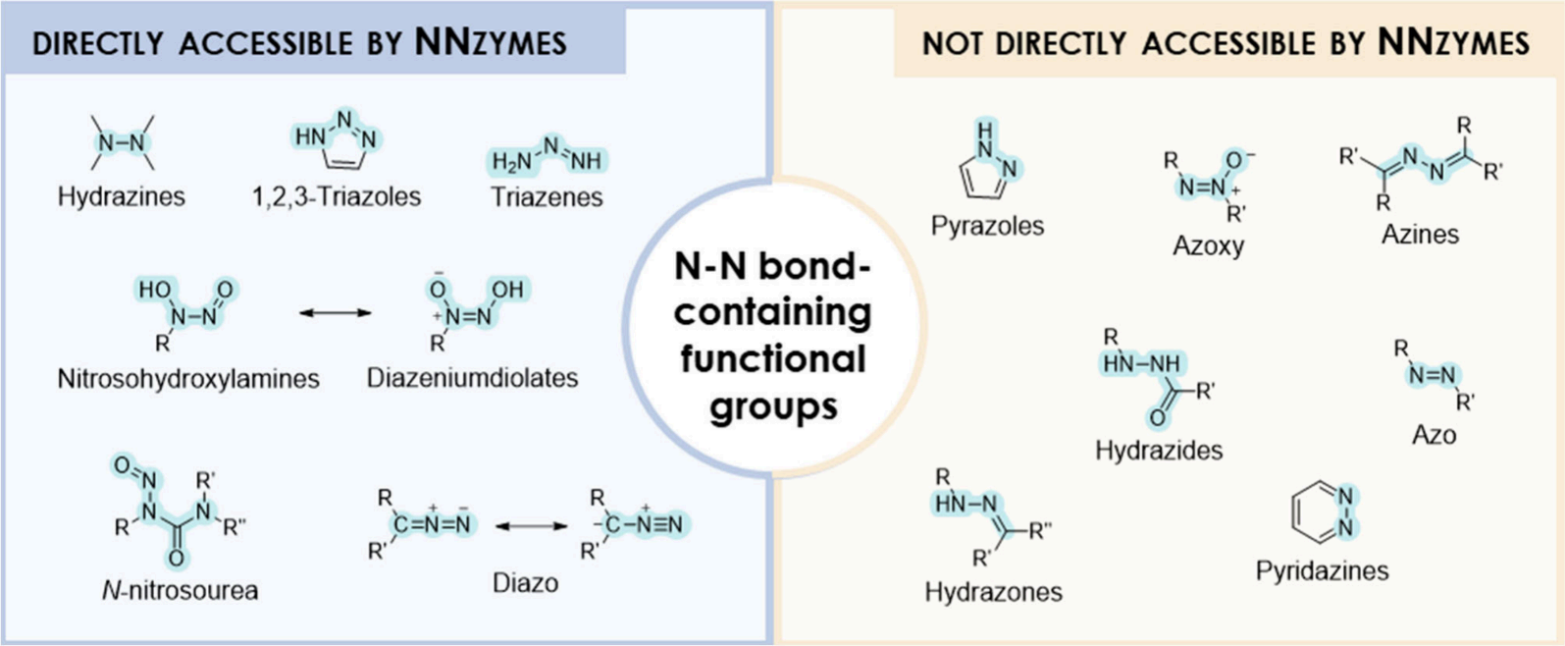
N–N bond-containing functional groups found in natural products (NPs). The blue background represents the functional groups that can be or are speculated to be constructed by nitrogen–nitrogen bond-forming enzymes (NNzymes). The orange background highlights remaining N–N bond-containing functional groups that can be found in NPs, but for which no corresponding NNzymes have yet been identified.

Together, these FDA approved drugs and NPs contain a number of different N–N functionalities, including: hydrazines (R_2_N-NR_2_), hydrazides (R_2_N–N(R)C(=O)R), hydrazones (R_2_C=N-NR_2_), azines (R_2_C=N–N=CR_2_), diazo- (R_2_C=N^+^=N^–^), azo- (RN=NR), azoxy- (RN=N^+^(O^–^)R), nitrosamines (R_2_N–N=O), triazenes (R_2_N–N=N-R) and aromatic heterocycles. However, direct access to some functionalities have not been associated with an NNzyme (Figure 2).^3,25^

The formation and cleavage of N–N bonds represent a fundamental biogeochemical process.^26^ Caranto et al. proposed that N–N bond-forming enzymes (NNzymes), which are involved in the biosynthesis of secondary metabolites, including characterized NPs, may have evolved from enzymes that participate in the nitrogen cycle.^27^ For an in-depth analysis of enzymes involved in the biosynthesis of primary nitrogen cycle metabolites, including nitrate, nitrite, nitric oxide and nitrous oxide, readers are encouraged to consult recent reviews.^28−30^

The growing interest in compounds containing the N–N motif has led to a dedicated and ongoing investigation into the synthesis of these molecules.^31^ Although accessible to organic chemists through the use of hydrazine and its derivatives, the hazardous and explosive nature of these substances makes them challenging to use in industrial applications and negatively impacts the environment.^32^ Instead, the direct coupling of nitrogen atoms, rather than hydrazine incorporation into a molecule, represents a highly efficient method that gives greater freedom when constructing molecules with N–N bonds. However, the synthetic challenge associated with the coupling together of two nitrogens to form an N–N bond can be attributed to the high nucleophilicity of the atoms involved. Therefore, the majority of the coupling methods rely on nitrogen activation to increase the electrophilicity of one nitrogen making coupling feasible.^33^ Examples of methods that achieve this nitrogen activation are electrochemistry,^34^ metal catalysis^35^ and oxidative or reductive transformations^36−41^ that have been developed to access a rich variety of molecules with N–N bonds. Despite the efforts made to improve classic organic synthesis methods, several challenges must be overcome, including product loss, low yields, poor accessibility of intermolecular reactions, low enantio- and diastereoselectivity, hetero- and homocoupling selectivity and the use of hazardous chemicals.^42,43^

A suitable solution to these challenges is to employ the use of enzymes as biocatalysts. Natural NNzymes have adopted methods to overcome this thermodynamic obstacle, resulting in the occurrence of N–N bond-containing functional groups in numerous secondary metabolites. The advent of genome mining techniques has greatly facilitated the exploration of the biosynthetic gene clusters (BGCs) responsible for the production of these metabolites. The enzymatic machinery responsible for N–N bond formation is gradually being elucidated within the clusters, leading to the discovery of an increasing number of NNzymes. Thus, a deeper understanding of the enzymatic machinery responsible for N–N bond formation in biosynthetic pathways and the development of NNzymes into broadly applicable biocatalysts could facilitate the creation of efficient, cost-effective, and more sustainable methods for the synthesis of N–N bond-containing compounds. It is therefore necessary to elucidate the functional and structural properties of these enzymes to expand their biocatalytic capabilities.

In recent years, a number of reviews have provided an overview of N–N bond formation in NP biosynthesis and summarized emerging NNzymes and their (proposed) enzymatic mechanisms.^3,11,25,44−48^ However, since the last review on (bio)synthetic routes to N–N bonds by the Ryan group was published in 2022,^25^ a plethora of novel N–N bond-containing NPs and their biosynthetic machinery have been elucidated. Importantly, more detailed information on the underlying enzymatic mechanisms and structural features of several NNzymes has also been unraveled since then. Therefore, this review aims to summarize the most recent understanding of the enzymatic and chemical mechanisms underlying N–N bond formation in NP biosynthesis and to provide a comprehensive overview of both characterized NNzymes and hypothetical biocatalysts with putative N–N bond forming activity. The enzymes are classified according to the functional groups they generate within the biosynthetic pathways of different NPs. Furthermore, a special emphasis is placed on NNzymes from a biocatalytic perspective, aiming to develop more sustainable methods for N–N bond formation. The classical synthetic pathways toward these functional groups can be found in Section 2. Section 3 covers the general reaction mechanisms and diversity of NNzymes, and Section 4 focuses on the biosynthesis of specific functional groups. Furthermore, Section 5 addresses the major challenges currently faced by classical synthetic approaches, with a particular emphasis on the potential for NNzymes to excel in areas where current limitations could be overcome.

## 2 Overview on Conventional Strategies to Access N–N Bond-Containing Functional Groups

As highlighted in this review, the construction of N–N bond-containing molecules in nature is a fascinating topic, especially in terms of how biological systems ingeniously overcome the high electronegativity of nitrogen atoms to create a diverse array of N–N bond-containing functional groups. While several evolved strategies for forming N–N bonds in nature appear to mirror known synthetic methodologies, others seem to diverge from these conventional routes. Thus, a thorough understanding of the biosynthetic processes is fundamental to advancing our knowledge of chemical bonding and reactivity, as the N–N bond displays properties that are distinct from those of the C–C and C–N bonds,^49^ and may also pave the way to the development of novel selective routes to N–N bond-containing molecules. This section of the review provides a general overview of the diverse N–N bond-containing functional groups and the conventional methods for their synthesis. Given the importance of biosynthetic N–N bond formation via enzymatic catalysis, we focus primarily on the synthesis of N–N bonds, which can also be accessed by NNzymes. For a more detailed discussion of the synthetic approaches, readers are directed to recent reviews.^11,25,28,31,33,44,50−52^

**Scheme 1:**
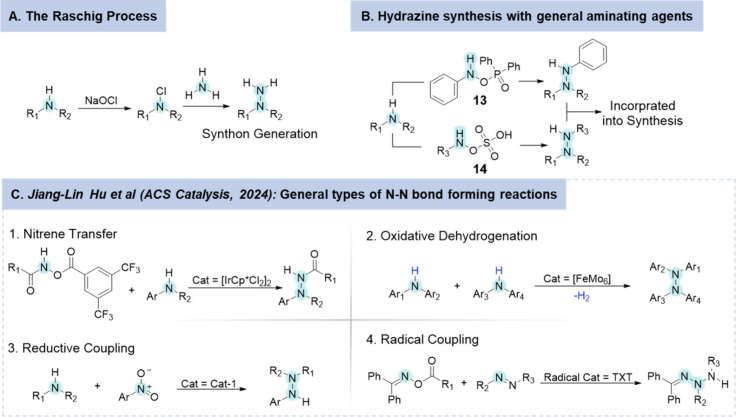
Catalytic Approaches for Creating an Electrophilic Partner for Forming Nitrogen–Nitrogen (N–N) Bonds **A**. The Raschig process **B**. Overview and classification of the methods used to form N–N bonds via activation of an electrophilic partner. **C**. Review of general types of N–N bond forming reactions from Jiang-Lin Hu et al.^33^ Noted reactions: **C1** nitrene transfer,^56^**C2** oxidative dehydrogenation,^57^**C3** reductive coupling,^58^ and **C4** radical coupling.^59^ Unless otherwise indicated, R represents an alkyl or aryl (Ar) group.

There are a number of potential retrosynthetic approaches that can be employed to access molecules containing N–N bonds (Scheme 1). The most frequently used method in the synthesis of N–N bond-containing molecules occurs from the functionalization and subsequent incorporation of the hydrazine synthon (Scheme 1A).^50^ Formed through the Raschig process, which has been modified over the past 100 years, these synthons provide access to a wide variety of N–N functional groups but are associated with significant toxicological concerns.^53^ The implementation of a hydrazine synthon as an indirect solution will not be discussed herein. Instead, the preactivation of an amine via an electrophilic partner, together known as an aminating reagent, allows for efficient linkage of two nitrogen atoms.^25^ Typical strong aminating agents such as *O*-diphenylphosphinyl-hydroxlamine (**13**)^54^ and hydroxylamine *O*-sulfonic acid (**14**)^55^ and have been shown to aminate a wide variety of amines (Scheme 1B).

A recent review by Jiang-Lin Hu et al.^33^ highlighted a variety of other catalytic approaches for creating an electrophilic partner for forming nitrogen–nitrogen (N–N) bonds (Scheme 1C). This included: nitrene transfer, oxidative dehydrogenation, reductive coupling, radical coupling and cycloadditions.^33^ The latter will not be further discussed herein due to the lack of enzyme-mediated routes.

Nitrene-transfer reactions work by generating highly reactive electrophilic metal–nitrene species from metal catalysts and nitrene precursors.^60^ These reactions provide access to a diverse range of *N*-aryl and alkyl hydrazides through the use of a variety of transition metal (TM)-based organometallic and inorganic complexes, including those containing Rh, Ir, Ni, Fe, Cu, and Ag.^61^ This methodology is applicable not only to relatively simple amines but also to complex natural molecules such as brucine or quinine.^62^

Oxidative dehydrogenation is the most direct and efficient strategy to perform a homo or hetero N–N bond formation.^63^ This process can be conducted with the use of transition metal catalysts, including complexes of Fe, Cu, and Co in conjunction with oxidants (e.g., H_2_O_2_), or radical initiators, such as azobis(isobutyronitrile) (AIBN). Copper catalysis has been particularly useful due to its versatility in aerobic oxidation reactions. An alternative approach to this reaction employs electrochemical methods, photoredox catalysis, and a hypervalent halide-mediated functionalization.^33^ This type of reaction allows for the formation of a wide range of products, including azo dyes, azines, carbazoles and heterocyclic compounds (pyrazoles, triazoles), while maintaining good functional group compatibility.^64^

Reductive coupling relies on nitroarenes, which are readily accessible compounds that act as direct aminating reagents and reducing agents. Although reductive coupling has more limitations than the first two types of reactions, due to the orthogonal or complementary reactivity of the catalytic complexes used, it can be combined with other types of catalysis (e.g., transition metals or biocatalysts) to achieve more sophisticated transformations. It has been demonstrated that reductive cyclization (heterocyclization) of benzamidines or 2-nitrobenzaldehydes, whether intramolecular or intermolecular, respectively, results in the effective conversion of both into 2*H*-indazoles.^52^ This type of reaction requires the use of redox couples of Bi^65^ or P,^58^ or, alternatively, Cu-based catalysts, which can also be used for condensation/cycloaddition cascades.^33^ Radical coupling methods are less common but involve the generation of nitrogen-centered radicals, which can combine to form N–N bonds. This approach is often initiated photochemically or by using radical initiators. This metal free method allows use of a variety of bifunctionalized oxime ester reagents.

The increasing number of methods for N–N bond formation highlights the growing interest in this area of chemistry. However, many of these approaches face challenges related to atom economy, cost efficiency, and sustainability, with limited consideration for achieving high levels of stereo-, chemo- and regioselectivity. Furthermore, many existing methods rely heavily on transition metal catalysis and harsh reaction conditions. This section will explore the chemical synthesis of specific N–N functionalities, emphasizing their significance and identifying potential areas where NNzymes could offer improvements over conventional, more demanding synthetic N–N bond forming routes.

### 2.1 Hydrazines, Hydrazides, and Hydrazones

Hydrazines (R_2_N–NR_2_) constitute a class of compounds characterized by the presence of two nitrogen atoms linked via a covalent bond, with one to four alkyl or aryl substituents. Linear and cyclic hydrazine derivatives, such as hydrazides R_2_N–N(R)C(=O)R and hydrazones (R_2_C=N–NR_2_), are extensively employed in the pharmaceutical, agrochemical, polymer and dye industries, facilitating a range of chemical processes. While nonderivatized hydrazines are typically not found in the final structure of natural products due to their high reactivity, they are widely utilized in biosynthetic pathways to form an array of N–N bond-containing natural products. Examples include, pyrazomycin (**1**),^8^ s56-p1 (**15**),^66^ formycin (**16**)^67^ and the triacsins (**17**).^68^

As previously mentioned, the Raschig process has been used since the start of the 20th century to synthesize hydrazine although the toxic nature of the process makes it unfavorable. Due to its nucleophilicity, hydrazine reacts with aldehydes and ketones in organic solvents, such as ethanol, methanol, and butanol to form hydrazones,^69^ key intermediates in many well-known synthetic reactions including the Wolff–Kishner reduction,^70^ the Wharton reaction^71^ and the Shapiro reaction.^72^ It can easily decompose into N_2_ gas and has been a part of the total synthesis of many molecules including scopadulcic acid B^73^ and dysidiolide.^74^

Hydrazine itself, along with its synthesis, is limited by issues of safety, sustainability and selectivity.^32,75^ Biocatalysis presents a promising alternative, offering the potential to overcome these limitations. Several N–N bond forming enzymes have already been identified, such as the cyclic hydrazine forming piperazate synthase (PZS) KtzT^76^ and its homologues^77−80^ being among the most extensively studied. For linear hydrazines, the hydrazine synthetase (HS), PyrN^81^ and its homologues^82^ have gained significant attention for their role in N–N bond formation at the initial stages of NP biosynthesis. The nonderivatized N–N bond is subsequently subjected to further transformations by other enzymes within the BGC. These enzymatic pathways will be discussed in Sections 4.1.2 and 4.4.

### 2.2 Azoxy and Azo Groups

Azoxy compounds represent a relatively minor category of naturally occurring molecules, sharing a common functional group and the general structural formula RN=N^+^(O^–^)R that represents the formally oxidized counterpart of the azo group (RN=NR).^83^ The azoxy moiety of secondary metabolites endows them with the capacity to damage DNA, a property that is perceived as both hazardous and carcinogenic. To date, azoxy compounds have been identified in a number of naturally occurring sources, including bacteria, fungi and plants.^84^ In the biosynthesis of **2**, an azoxy group was reported to be formed via an azo compound that is not present in the structures of the final secondary metabolites.^67^

The azo functional group is utilized ubiquitously in chemical synthesis such as in the Mitsunobu reaction where it forms part of the diethyl azodicarboxylate (DEAD) reagent, also as part of the radical initiator compound AIBN. Additionally, azo dyes, formed by azo coupling reactions, constitute for 60–70% of all dyes used in the food and textile industries.^85^

The most common method for the preparation of aryl azo compounds employs the use of highly energetic diazonium salts.^86^ The azoxy and azo groups can also be accessed by oxidative cross-coupling of anilines, or by reductive coupling of nitrobenzene compounds.^83,87^ Traditional oxidative coupling methods frequently employ environmentally unfriendly oxidants, including peroxides, copper or silver-based salts, and transition metal-based catalysts, such as ruthenium complexes.^88^

Given the significant importance of these functionalities, it is necessary to develop alternative sustainable methods for their synthesis. It has recently been reported, that oxidative coupling can be performed enzymatically using AzoC,^89^ which is not an NNzyme but allows the formation of an N–N bond via a spontaneous radical mechanism. Additionally, progress was made in the reductive coupling of nitrobenzene compounds using a photoenzymatic approach, which highlights the importance of transitioning to biotechnological methods.^90^

### 2.3 Diazo Group

Diazo compounds (R_2_C=N^+^=N^–^), though relatively rare in nature, play a significant role in secondary metabolite biosynthesis due to their unique chemical properties. In the 1950s, the first diazo compounds discovered included modified α-amino acids like azaserine (**18**), 6-diazo-5-oxo-l-norleucine (DON, **19**) and azotomycin, which were obtained from different *Streptomyces* strains.^91−93^ Since then, research on diazo compounds has continued, resulting in the discovery of intriguing bioactive molecules like kinamycin D (**20**) and lomaiviticin A (**21**). Studies on these diazofluorene-based antitumor antibiotics have revealed the crucial role of diazo groups in their biological activity.^94^ These compounds are best known for their involvement as versatile intermediates in modern synthetic organic chemistry, such as in the Bamford–Stevens,^95^ Doyle–Kirmse^96^ and the Büchner–Curtius–Schlotterbeck^97^ reactions.^98^ They can also be generated *in situ* from precursors such as hydrazones.^99^

Due to their ylide nature, the thermal stability, reactivity and liability toward strong acids is an important concern when choosing to use diazo compounds in synthetic routes. Their reactivity can be tuned however by changing the substituent on the diazo carbon. For simple aliphatic and electron donating groups, the functional group is unstable but with aromatic and substituted electron withdrawing groups, it becomes more stable. Although they are incredibly useful intermediates, their safety risk means that few are made commercially available. Especially for the highly hazardous and explosive diazomethane which when used in industry requires continuous production and consumption.^100^ Therefore, in situ consumption in a continuous process is key to maintaining safety and also utilizing the functionality. The biocatalytic pathways present here-in allow for safe control of these intermediates while accessing a diverse range of chemistries.^101^

### 2.4 *N-*Nitroso Group

*N*-nitro groups can be classified into several types based on the atoms to which the nitroso group (NO) is directly attached.^102^ The present review will focus on *N*-nitrosamine compounds, in which the *N*–NO bond formation is catalyzed by an NNzyme. *N*-nitroso compounds can be further classified as *N*-nitrosamines (R_1_N(−R_2_)–N=O), *N*-nitrosohydroxylamines (R_1_N(−OH)–N=O), and *N*-nitrosamides (R_1_C(=X)N(−R_2_)–N=O) with the derivatives, *N*-nitrosocarbamates, *N*-nitrosoureas and *N*-nitrosoguanidine. Among the various biologically active NPs, the *N*-nitroso compounds represent the largest group, including l-alanosine (**22**), gramibactin (**23**), (−)-fragin (**24**), and chalkophomycin (**25**) due to their ability to coordinate metals and act as metallophores.^103^

*N*-nitroso compounds have been demonstrated to possess mutagenic and carcinogenic properties, whereby they can be bioactivated, forming a carbonium ion that facilitates alkylation of diverse cellular macromolecules.^104^ Consequently, they represent a promising class of compounds for potential exploitation in chemotherapy.^105^ One prominent example is streptozocin (**5**), which belongs to the *N*-nitrosamides group and exerts its activity by generating electrophilic DNA alkylating agent and nitric oxide. The conventional synthetic methodologies for accessing *N*-nitroso compounds typically employ a range of nitrosating reagents, including nitrosyl halides (e.g., NOCl, NOBr), nitrogen oxides (e.g., NO, N_2_O_3_, N_2_O_4_), nitro compounds (e.g.,CH_3_NO_2_), and nitrite salts (e.g., alkyl nitrites, NaNO_2_); however, a majority of them are toxic.^102,106^

The discovery of *N*-nitrosamine impurities in active pharmaceutical ingredients (APIs) has become a global concern. In 2021, the European Medicines Regulatory Network established the Nitrosamines Implementation Oversight Group (NIOG) to oversee risk and implement mitigation strategies. One key approach involves eliminating these toxic nitrosating agents by modifying solvents or synthetic routes. Biocatalysis presents a promising alternative, offering a more sustainable synthesis pathway that avoids the use of nitrosating agents altogether, thus providing enhanced control and reducing the risk of carcinogenic impurities in pharmaceutical products.^107^

### 2.5 Triazenes and Triazoles

The triazenes are linear molecules comprising three adjacent nitrogen atoms (RN=N–NR_2_). Aromatic compounds containing triazenyl groups, such as 1,2,3-triazole, have a profound impact on the fields of synthetic organic and medicinal chemistry.^108^ In contrast, the chemistry of vinyl and alkynyl triazenes was previously an area of limited investigation. Nowadays, it has become evident that vinyl and alkynyl triazenes are highly intriguing compounds with distinctive reactivity.^109^ Triazenes have been investigated for their potential anticancer properties, employed as protecting groups in NPs synthesis and combinatorial chemistry, and utilized to generate novel heterocycles. Their biological activity is derived from their capacity to form diazonium salts, which are capable of alkylating DNA. Additionally, triazenes can be transformed into a range of reactive groups following the application of suitable reagents.^110^ Triazenes are easily synthesized from readily available anilines or alkyl azides. Dialkyl triazenes can be obtained from the reaction of an alkyl azide with the appropriate Grignard or alkyl lithium reagent.^111^ It is noteworthy that most triazene syntheses were optimized before the 1930s and that some of the most useful preparative routes have hardly changed in the last 100 years since their initial discovery. However, this longevity has also meant that the sustainability of the process has not been reassessed.^112^

Given the significance of triazoles, the synthesis of these compounds has been the subject of considerable research. Following the advent of the 1,3-dipolar cycloaddition between azide and terminal alkyne, also known as the Watson cycloaddition,^113^ the Cu-catalyzed azide–alkyne cycloaddition reaction (CuAAC), as proposed by Sharples, became the predominant method for synthesizing 1,2,3-triazoles, more commonly referred to as “click chemistry”.^114^ Although enzymatic methods are underrepresented in heterocyclic chemistry, there are a few examples of newly discovered BGCs that have the potential to be explored in this field, which are further discussed in Sections 4.4 and 4.5.

Traditional synthetic approaches to N–N bond formation of these functional groups often involve harsh conditions, toxic reagents, and a tendency toward undesirable selectivity. This presents significant obstacles for achieving efficient, controlled N–N coupling reactions. NNzymes, however, offer a promising alternative by circumventing many of the challenges while opening new avenues for the production of complex N–N functional groups. Given this, a thorough understanding of the biosynthetic processes is fundamental to advancing our knowledge of chemical bonding and reactivity. The integration of biocatalysis into synthetic methodologies holds the potential to revolutionize N–N bond construction, providing a more sustainable and selective approach to accessing these unique functional groups.

## 3 Biosynthesis of N–N Bond-Containing Functional Groups Catalyzed by NNzymes: General Reaction Mechanisms and Diversity

A diverse array of N–N bond-containing functional groups are biosynthesized by known or hypothetical NNzymes, for which a comprehensive overview is given in Table 1, categorized according to the functional group they construct. In general, NNzymes can be divided into heme-dependent, heme-oxygenase–like diiron oxidase and oxygenase (HDO), and cupin-dependent biocatalysts, but also biocatalysts that belong to uncharacterized structural groups. Among the relatively small but well-established group of heme-dependent enzymes, the most well-known representative is the PZS KtzT.^76^ In contrast, the heme-like proteins lack heme occupancy but coordinate iron in the vicinity of their active site. The cupin fold is found in one of the most versatile protein families and has been linked to the formation of N–N bonds in numerous examples. The majority of the cupin-dependent biocatalysts contain iron in their active site. However, the cupin domain can also coordinate metal ions such as zinc, copper, cobalt or manganese.^115^ One particularly illustrative example is the protein SznF/StzF from *Streptomyces achromogenes* subsp. *streptozoticus*, which possesses one HDO central domain and a C-terminal monoiron cupin domain.^116^ Moreover, for the majority of NNzymes or hypothetical enzymes involved in N–N bond formation, no structural information is currently available. Nevertheless, these enzymes are involved in highly intriguing biosynthetic processes that make them a subject of considerable interest. Despite the remarkable diversity of N–N bond-containing NPs, NNzymes have been identified for the construction of specific functionalities, including hydrazines, diazo- and nitroso- groups, triazenes and triazoles (Figure 2). This review will provide a detailed description of these biocatalysts.

**Table 1:** List of Putative and Confirmed NNzymes Covered in This Review

**Protein name**	**Accession number**	**Natural product**	**Organism**	**Functional group**	**MiBig Entry**	**Putative or ConfirmedNNzyme**^a^	**ref**
**Intramolecular hydrazine group formation**							
KtzT	UniProt ID: A8CF72	kutznerides (**26**)	*Kutzneria* sp. 744	Hydrazine	BGC0000378	Confirmed (*in vitro*)	(76)
SfaC	UniProt ID: D3U9Y3	sanglifehrin A	*Streptomyces flaveolus*	Hydrazine	BGC0001042	Confirmed (*in vitro*)	(120)
HmtC	UniProt ID: D9WMP1	himastatin	*Streptomyces himastatinicus* ATCC 53653	Hydrazine	BGC0001117	Confirmed (*in vitro*)	(80)
PadO	UniProt ID: U5YN79	padanamides	*Streptomyces* sp. RJA2928	Hydrazine	-	Confirmed (*in vitro*)	(80)
Luz13	NCBI ID: UKU09931.1	luzopeptins	*Actinomadura luzonensis*	Hydrazine	-	Confirmed (*in vivo*)	(121)
Kor13	-	korkomicins	*Micromonospora* sp. ATCC 55011	Hydrazine	-	Putative	(121)
MtPizS	NCBI ID: WP_230415462	unknown	*Micromonospora tarapacensis*	Hydrazine	-	Confirmed (*in vitro*)	(77)
SpPizS	NCBI ID: WP_111501290	unknown	*Streptacidiphilus pinicola*	Hydrazine	-	Confirmed (*in vitro*)	(77)
PipS	NCBI ID: WP_030722408.1	unknown	*Streptomyces griseus* subsp. *griseus*	Hydrazine	-	Confirmed (*in vitro*)	(79)
PAI2	UniProt ID: A0A5B7 V5A7	unknown	*Streptomyces* sp. YIM 121038	Hydrazine	-	Confirmed (*in vitro*)	(78)
MatF	NCBI ID: WP_240489931	matlystatins	*Actinomadura atramentaria DSM 43919*	Hydrazine	BGC0001443	Confirmed (*in vitro*)	(80)
XF36	UniProt ID: A0A0M4QM67	gerumycins	*Pseudonocardia* sp. HH130629-09	Hydrazine	-	Confirmed (*in vitro*)	(80)
**Intermolecular ATP-dependent hydrazine group formation**							
Spb40	UniProt ID: A0A1L7NQI6	s56-p1 (**15**)	*Streptomyces* sp. SoC090715LN-17	Hydrazine	BGC0001764	Confirmed (*in vivo*)	(66)
Tri28	Uniprot ID: I2MZC8, UniProt ID: A0A4P8XS63	triacsins (**17**)	*Streptomyces tsukubensis Kitasatospora aureofaciens*	Hydrazine	BGC0001983 (*Kitasatospora aureofaciens*)	Confirmed (*in vitro*)	(68)
Aza12/AzaE	UniProt ID: A0A1G6ZJC9	azaserine (**18**)	*Glycomyces harbinensis* ATCC 43155	Hydrazine	-	Confirmed (*in vivo*)	(122−124)
SFaza12/AzsN	NCBI ID: WP_108953673.1	azaserine (**18**)	*Streptomyces fragilis*	Hydrazine	-	Confirmed (*in vitro*)	
PyrN/PrfJ	UniProt ID: A0A516ELE7	pyrazomycin (**1**)	*Streptomyces candidus*	Hydrazine	-	Confirmed (*in vitro*)	(8)
ForJ	Uniprot ID: QTK22492, NCBI ID: WP_051869537.1 and WP_012180808.1	formycin (**16**)	*Streptomyces kaniharaensis*, *Streptomyces resistomycificus*, *Salinispora arenicola CNS-205*	Hydrazine	-	Confirmed (*in vitro*)	(67)
Apy9	NCBI ID: BDC79915	actinopyridazinone A, B (**55**, **54**)	*Streptomyces* sp. MSD090630SC-05	Hydrazine	-	Confirmed (*in vitro*)	(125)
Por11	NCBI ID: WP_057724816.1	unknown	*Pseudomonas orientalis*	Hydrazine	-	Confirmed (*in vivo*)	(125)
**Intermolecular nitrite-dependent hydrazine group formation**							
FzmP	UniProt ID: U5YN85	fosfazinomycin (**65**)	*Streptomyces* sp.	Hydrazine	BGC0000937	Putative	(126)
KinJ	UniProt ID: A0A385LMJ2	kinamycins (**20**)	*Streptomyces murayamaensis*	Hydrazine	BGC0000236	Putative	(45)
Alp1J	UniProt ID: Q1RQT7	prekinamycin	*Streptomyces ambofaciens* strain ATCC 23877	Hydrazine	-	Putative	(182)
Lom29	UniProt ID: A0A059UDU0	lomaiviticins (**21**)	*Salinispora tropica* CNB-440 *Salinispora pacifica* DPJ-0016 and DPJ-0019	Hydrazine	BGC0000241 (*Salinispora tropica*) BGC0000240 (*Salinispora pacifica*)	Putative	(128), (129)
ORF38	Uniprot ID: F6K0Z1	fluostatins (**71**)	uncultured bacterium BAC AB649/1850	Hydrazine	BGC0001596 (*Streptomyces albus*) BGC0000223 (uncultured bacterium) BGC0001904 (*Micromonospora rosaria*)	Putative	(129)
**Azoxy functional group via hydrazine intermediates**							
VlmO	UniProt ID: Q84F35, Uniprot ID: E4N6B1	valanimycin (**2**)	*Streptomyces viridifaciens Kitasatospora setae* (strain ATCC 33774)	Hydrazine	-	Confirmed (*in vitro*)	(130)
KaO	NCBI ID: BBC93011.1	KA57-A	*Streptomyces rochei* 7434AN4	Hydrazine	-	Putative	(130)
ElaO	NCBI ID: WP_189274298.1	elaiomycins	*Streptomyces atratus* NRRL-16927	Hydrazine	-	Putative	(130)
AzdO	BGC ID: BGC0002805	azodyrecins (**78**)	*S*. *mirabilis* P8-A2	Hydrazine	-	Confirmed (*in vivo*)	(131)
Ady6	NCBI ID: BDI55413.1 and BDI55430.1	azodyrecins (**78**)	*Streptomyces* sp. A1C6 and *Streptomyces* sp. RM72	Azoxy	-	Putative	(132)
**Diazo functional group**							
CreM	UniProt ID: A0A0K2JLU1	cremeomycin (**3**)	*Streptomyces cremeus*	Diazo	BGC0001295	Confirmed (*in vivo* and *in vitro*)	(133)
AzpL	NCBI ID: WP_157538045.1	alazopeptin (**86**)	*Kitasatospora azatica Streptacidiphilus griseoplanus*	Diazo	BGC0002536 (*Kitasatospora azatica*) BGC0002457 (*Streptacidiphilus griseoplanus*)	Confirmed (*in vivo*)	(134)
Aha11	NCBI ID: UMM61389.1	tasikamides A-C (**88**–**90**)	*Streptomyces tasikensis*	Diazo	BGC0002661	Confirmed (*in vitro*)	(135)
SpiA7	NCBI ID: WP_189300626.1	spinamycin (**93**)	*Streptomyces albospinus*	Diazo	-	Confirmed (*in vitro*)	(136)
Pzm18	NCBI ID: UUJ74630.1	penzoemycins A and B (**98**, **99**)	*Streptomyces* sp. SCSIO 40020	Diazo	-	Putative	(137)
AvaA6	NCBI ID: BDI54813.1	avenalumic acid (**95**)	*Streptomyces* sp. RI-77	Diazo	-	Confirmed (*in vitro*)	(138)
CmaA6	Uniprot ID: W5W4E6	*p*-coumaric acid (**104**)	*Kutzneria albida* DSM 43870	Diazo	-	Confirmed (*in vitro*)	(139)
NapB4	UniProt ID: A7KGZ4, Uniprot ID: A7KH22	azamerone (**84**)	*Streptomyces aculeolatus Streptomyces* sp. CNQ-525	Diazo	BGC0001079 (*Streptomyces aculeolatus*) BGC0000652 (*Streptomyces* sp. CNQ-525)	Putative	(10)
**Nitrosamide (*****N*****-nitrosourea) functional group**							
SznF/StzF	UniProt ID: A0A411MR89	streptozocin (**5**)	*Streptomyces*. *achromogenes* subsp. *streptozoticus*	*N*-Nitroso-amides	BGC0002313 (SznF) BGC0002294 (StzF)	Confirmed (*in vitro*)	(116), (140)
**Nitrosohydroxylamine functional group**							
Aln *A*	Uniprot ID: A0A6B9JBV1	l-alanosine (**22**)	*Streptomyces alanosinicus*	*N*-Nitroso-hydroxylamines	-	Putative	(141), (142)
GrbD	UniProt ID: B1G5G9	gramibactin (**23**)	*Paraburkholderia graminis Paraburkholderia caledonica*	*N*-Nitroso-hydroxylamines	BGC0001999 (*Paraburkholderia graminis*) BGC0002563 (*Paraburkholderia caledonica*)	Putative	(143−145)
HamA and/or HamE	UniProt ID: A0A144VC93, UniProt ID: A0A1 V2W1F5	(−)-fragin (**24**) valdiazen (**116**)	*Burkholderia cenocepaci*a H111	*N*-Nitroso-hydroxylamines	BGC0001599	Putative	(146)
ChmM	UniProt ID: QNQ35080	Cu^II^-chalkophomycin (**25**)	*Streptomyces* sp. CB00271	*N*-Nitroso-hydroxylamines	-	Putative	(103)
**Triazene functional group**							
Tri17	UniProt ID: A0A7G3URI3, UniProt ID: A0A4P8XUW1	triacsins (**17**)	*Streptomyces tsukubensis* NRRL 18488 *Kitasatospora aureofaciens* ATCC 10762	Triazene	BGC0001983 (*Kitasatospora aureofaciens*)	Confirmed (*in vitro*)	(68)
**Triazole functional group**							
PtnB/8-AzgE	UniProt ID: A0A6G9KGS5/A0A7G3ZQC3	8-azaguanine (**126**)	*Streptomyces pathocidini* ATCC 14510	1,2,3-Triazole	BGC0002508	Putative	(147), (148)

a Confirmed activity refers to N–N bond formation catalyzed by the indicated enzyme, but has not necessarily been confirmed for the native substrate within the biosynthesis of the respective NP.

**Figure 3:**
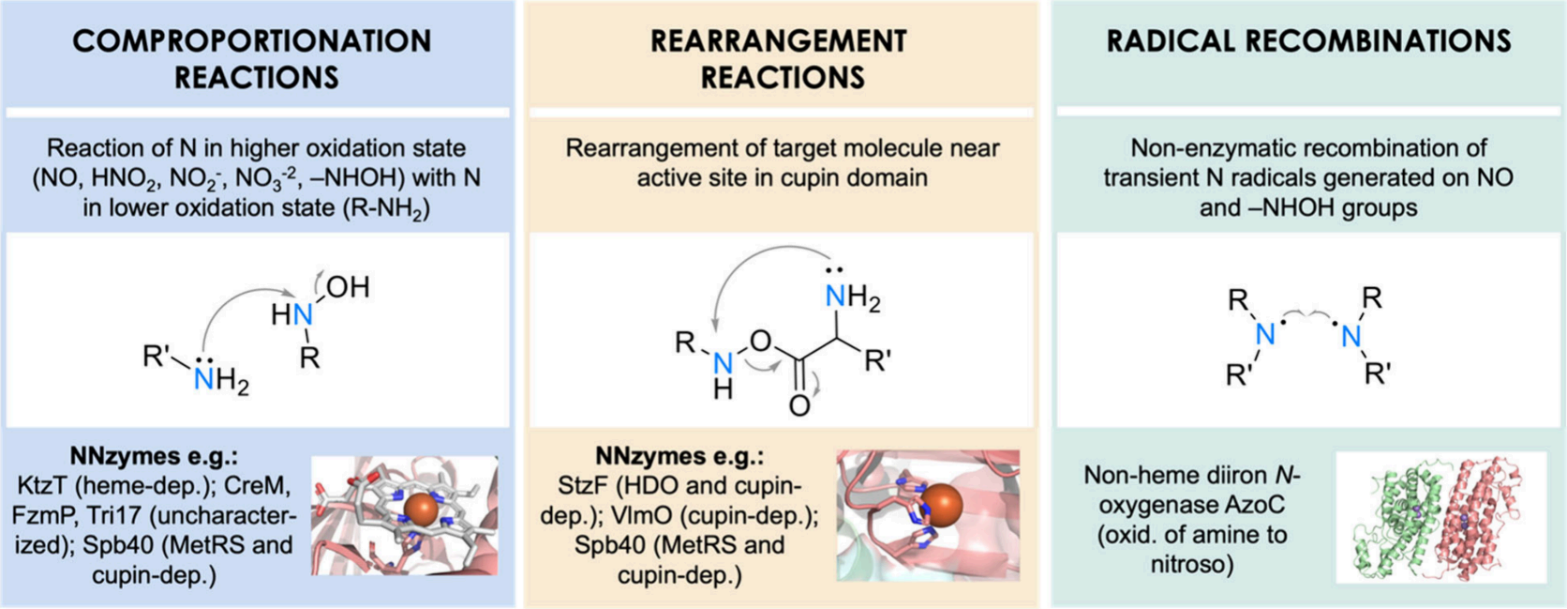
Three different mechanisms for the biosynthesis of N–N bonds and the corresponding NNzymes. Abbreviations: HDO, heme-oxygenase-like diiron oxidase and oxygenase; MetRS, methionyl-tRNA-synthetase-like.

The biosynthesis of these functional groups can be achieved by either an intra- or intermolecular mechanism. The nature of this mechanism can be determined by the presence or absence of an external nitrogen donor. In addition, numerous mechanisms of NNzymes involve the formation of an N–N bond through the addition of nitrogen oxide species, including nitric oxide (NO), nitrous acid (HNO_2_), nitrite (NO_2_^–^), nitrate (NO_3_^–2^), and the hydroxylamine group (−NHOH). These electrophilic moieties can be readily attacked by the second nucleophilic nitrogen atom of an amine group (R–NH_2_), in a more general class of comproportionation (synproportionation) reactions,^117^ thereby forming the N–N bond. Notable examples of NNzymes that exhibit this mechanism are KtzT, CreM, Tri28, Tri17, among others, which are discussed in the following sections. An additional core strategy that results in the formation of N–N bonds involves the rearrangement of the target molecule in the vicinity of the active site in the cupin domain, ultimately leading to the formation of an N–N bond. The most extensively studied representative catalyzing a rearrangement reaction is SznF/StzF, while Spb40 can be included in both of these two groups according to the proposed reaction mechanism (Scheme 5). Another approach to the biosynthesis of N–N bond-containing NPs involves the spontaneous recombination of transient nitrogen radicals without the involvement of NNzymes, as reported in the biosynthesis of azoxymycins,^118^ in which the nonheme diiron oxygenase AzoC (UniProt ID: A0A0K0PIV3) generates the nitroso precursors required for N–N bond formation (Figure 3).^119^ However, the formation of N–N bonds via a radical-mediated process will not be further discussed herein.

## 4 Functional Group Diversity Accessible to NNzymes

### 4.1 Hydrazine Functional Group

#### 4.1.1 Intramolecular Hydrazine Formation via Heme-Dependent Piperazate Synthases

**Scheme 2:**
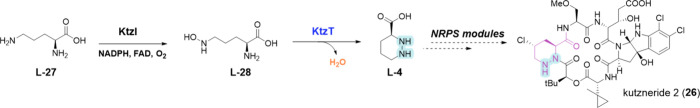
Biosynthesis of l-Piz (**L-4**) in *Kutzneria* sp. 744^76^

Piperazic acid (systematic name: (*S*)-hexahydropyridazine-3-carboxylic acid; **L-**Piz, **L**-**4**) is a noncanonical, secondary α-hydrazino acid containing a hydrazine bond in its 1,2-diazinane heterocyclic side chain. A large number of bioactive molecules are derived from the structural scaffold of Piz, which are found in hundreds of nonribosomal peptide synthetase (NRPS)-derived secondary metabolites,^11^ many of which exhibit potent biological activities, such as the anticancer agents himastatin,^149^ luzopeptins^150^ and depsidomycins,^151^ the antibiotics monamycin^152^ and matlystatin,^153^ or the antifungal kutznerides (**26**).^154,155^ Another example of Piz-containing biologically active compounds is the KRAS G12D inhibitor RMC-9805, which has recently been introduced into Phase 1 clinical trials.^156^ In 2012, the BGC of kutznerides was identified^157^ and it was found by Neumann et al. that an FAD-dependent *N*-hydroxylase KtzI (UniProt ID: A8CF85) is critical for the biosynthesis of **L-4** (Scheme 2). KtzI is active toward l-ornithine (**L-27**) to produce a hydroxylamine intermediate, *N*^5^-hydroxy-l-ornithine (**L-28**).^158,159^ The formation of the N–N bond remained elusive until Du et al. identified a heme b-dependent synthase KtzT (originally named *orf*4 in the kutzneride BGC) that constructs the hydrazine bond of l-Piz in *Kutzneria* sp. 744 (Scheme 2).^76^

More specifically, the KtzI-catalyzed “activation” of **L-27** yields **L-28**, which is the substrate for a ring-closing condensation catalyzed by KtzT, yielding the hydrazine bond in **L-4** (Scheme 2). This enzymatic pair of *N*-hydroxylating monooxygenase (NMO) and PZS is also widely distributed in other BGCs responsible for the biosynthesis of various Piz-containing molecules, with some notable KtzI-KtzT homologous pairs being SfaB (UniProt ID: D3U9Y2)-SfaC in the biosynthesis of sanglifehrin A,^160^ PadN (UniProt ID: U5YL02)-PadO in the biosynthesis of padanamides,^161^ HmtM (UniProt ID: D9WMQ4)-HmtC in the biosynthesis of himastatin,^162^ Kor17-Kor13 in the biosynthesis of korkomicins,^121^ and Luz17 (NCBI ID: UKU09924.1)-Luz13 in the biosynthesis of the luzopeptins.^121^ This enzymatic pair does not always appear as two distinct genes, as Hu et al. showed that naturally occurring didomain NMO-PZS chimeric enzymes exist which can produce **L-4** in actinobacteria and also *in vitro* from **L-27**.^120^ The discovery of the KtzI-KtzT pair in Piz monomer production has enabled the usage of targeted metabolomics,^163^ and genomic signature-based screening methods^164^ to identify novel Piz-containing natural products. Combined with specific product identification techniques like ^15^N NMR-based screening for Piz,^164,165^ these approaches have greatly simplified the identification of Piz-producing organisms and Piz-containing NPs, and already led to the discovery of previously unreported anticancer agents such as incarnatapeptin B^165^ and petrichorins.^163^

**Scheme 3:**
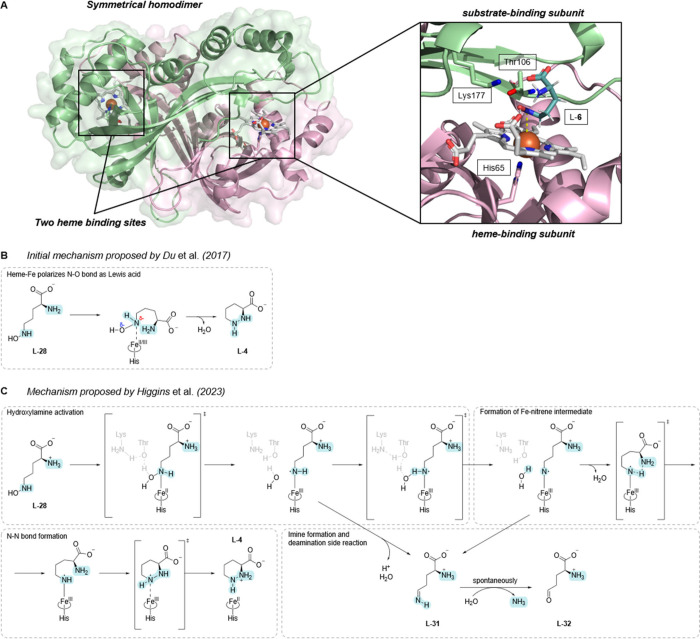
Detailed Prediction of the Reaction Mechanism **A**, AlphaFold3 model of homodimeric KtzT-heme with close-up of the active site highlighting catalytically relevant residues and docked *N*^5^-hydroxy-l-ornithine (**L-28**). The separate monomers are shown in green and pink. The heme b cofactor (white), heme-binding residue H65 (pink), proposed catalytic dyad (green) and **L-28** (cyan) are shown as sticks. The coordinated heme iron (orange) is shown as a sphere. **B**, Initial and **C**, updated proposed reaction mechanisms for PZS-catalyzed conversion of **L-28** to **L-4**.^76,79^ The lower scheme also illustrates the manner in which the imine formation pathway, observed for specific non-native substrates, would proceed with **L-28**.

Despite these advances, structural and mechanistic knowledge on how N–N bond formation is achieved by PZSs is still very limited. Size-exclusion chromatography experiments revealed that KtzT forms dimers in solution, and structural predictions suggest homodimers with two symmetrical active sites (Scheme 3A).^76^ From the analysis of the PZS consensus sequence and subsequent mutagenesis studies, Du et al. found the conserved residue H65 in KtzT to be crucial for heme binding and its catalytic activity (Scheme 3A).^76^ More specifically, they predicted the heme iron to act as a Lewis acid, inducing polarization of the hydroxylamine bond of the activated substrate **L-28** to allow a nucleophilic attack by the α-*N* on the δ-*N* to occur, eliminating water in the process (Scheme 3B). Our own studies identified the residue C197 in KtzT to form a dimer-linking disulfide bond, being at least partly responsible for dimerization of that specific PZS.^77^ However, the C197S mutation does not negatively influence the catalytic activity of KtzT, and notably, certain homologues, such as SfaC, naturally lack a cysteine residue in their C-terminal region.^120^ Thus, neither a cysteine at that position nor a disulfide bond are essential for functional PZS. Apart from the native substrate, KtzT has been shown to exhibit some promiscuity, being slightly active on **D-28** and *N*^4^-hydroxy-l-diaminobutanoic acid (**L-29**) as well, yielding **D-4** and 5-aza-l-proline (**L-30**), respectively.^76^ Enzyme profiling experiments conducted on KtzT revealed preferred optimal reaction conditions at 30 °C and low salt concentrations, and a pH optimum around 9, which potentially benefits deprotonation of the α-amine of the substrate, increasing nucleophilicity.^77^ Homologous PZS from *Micromonospora tarapacensis*, MtPizS (52% sequence identity to KtzT), *Streptacidiphilus pinicola*, SpPizS (59% sequence identity to KtzT), *Actinomadura atramentaria* DSM 9954, MatF and *Pseudonocardia* sp. HH130629-09, XF36, were studied *in vitro* as well and were shown to exhibit PZS activity on **L-28**.^77,80^ Recently, a more detailed prediction of the reaction mechanism emerged (Scheme 3C) when Higgins et al. solved the crystal structure of a PZS PipS from *Streptomyces griseus* subsp. *griseus* NRRL F-5144 (54% sequence similarity to KtzT).^79^

**Scheme 4:**
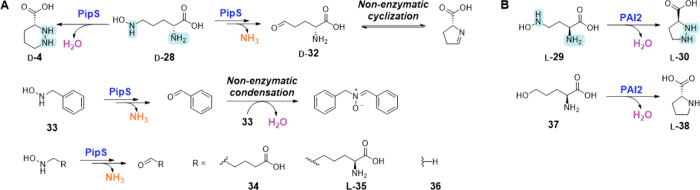
Overview of Nonnative PZS-Catalyzed Reactions **A**, Hydrazine synthase and dehydratase activity of PipS on non-native substrates. **B**, Hydrazine synthase and C–N lyase activity of PAI2 on non-native substrates.^76,78,79^

When combined with electron paramagnetic resonance (EPR) experiments and extensive quantum mechanics/molecular mechanics (QM/MM) simulations, the findings indicate a reaction mechanism in which a key radical Fe–N nitrene intermediate is formed. This increases the electrophilicity of the δ-*N*, facilitating nucleophilic attack by the α-amine as proposed in the previous study. Higgins et al. also highlight the involvement of a threonine-lysine catalytic dyad in the active site likely responsible for proton transfer and water elimination. Their studies also revealed a side-reaction occurring on certain non-native substrates, in which no hydrazine bond is formed, but where the nitrene intermediate spontaneously dehydrates to yield the corresponding imine (**L-31**), which further hydrolyzes to an aldehyde (**L-32**) in the aqueous reaction environment (Scheme 3C). For example, PipS was shown to exhibit solely dehydratase activity on *N*-benzylhydroxylamine (**33**), 5-hydroxyamino-pentanoic acid (**34**), *N*^6^-hydroxy-l-lysine (**L-35**), and *N*-methylhydroxylamine (**36**), while it exhibits both hydrazine synthase and dehydratase activities on **D-28** yielding **D-4** and **D-32**, respectively (Scheme 4A).^79^ These recent findings on the PZS family may help to understand these biocatalysts and enable the exploitation of their potential for biotechnological applications.

PAI2 is another heme-dependent KtzT homologue with 65% sequence similarity. It is not to be confused with the gene product of *pai*B, “PAI2”, which is a transcription regulator protein in *Bacillus subtilis* (UniProt ID: P21341) and *Bacillus stearothermophilus* and is a structural homologue (PDB ID: 2OL5) to PZS. The corresponding gene was identified in the genome of *Streptomyces* sp. YIM 121038, and has been sufficiently characterized.^78^ Its PZS activity on **L-28** was shown to be higher than that of KtzT, and it was also shown to convert **L-29** to a five-membered pyrazolidine-containing cyclic α-amino acid **L-30** (Scheme 4B). In addition to that, it has been reported to catalyze the analogous formation of a C–N bond in (*S*)-2-amino-5-hydroxypentanoic acid (**37**) to give l-proline (**L-38**), though not catalyzing the respective reactions in (*S*)-2-amino-6-hydroxyhexanoic acid and (*S*)-2-amino-7-hydroxyheptanoic acid. To date, its unique C–N bond-forming activity has not been further investigated and has not been reported for other PZS. In addition to the reported LC-MS trace of the enzymatic reaction, NMR spectra of the postulated product would be required to more conclusively prove its C–N lyase activity. Through structural investigations on a homology model of PAI2 and subsequent mutagenesis studies, it was found that residue A104 is essential for the catalytic activity on **L-28**, likely being involved in binding the substrate through hydrophobic interactions.

Apart from **L-4**, its congeners such as 5-hydroxy-, 5-chloro-, and 1,6-dehydro-5-hydroxy-piperazate are widely incorporated into various secondary metabolites such as svetamycins.^11,166^ It has been demonstrated that the halogenase KthP (UniProt ID: W7T5C7) is capable of installing a *C*^5^ chlorine on l-Piz (**L-4**), dependent on the presence of a piperazyl-*S*-thiolation domain.^167^ Furthermore, HmtN (UniProt ID: D9WMQ6), a heme-dependent cytochrome P450 monooxygenase, has been demonstrated to play a role in the subsequent hydroxylation of **D-4**.^162^ Another example of **L-4** modification involves the multifunctional cytochrome P450 Luz26 that catalyzes an unusual C–N bond desaturation, leading to hydrazone formation from a hydrazine intermediate in the biosynthesis of luzopeptin A.^121^

The conventional synthetic route to **L-4** and other cyclic α-hydrazino acids is notably complex. For instance, one of the protocols utilizing commercially available diethyl malonate, allyl or homoallyl bromides, and azodicarboxylates as starting materials can access the final product with eight to nine steps and an overall yield of 13–34%.^168^ However, the synthesis requires the protection of functional groups, and the use of highly reactive reagents such as osmium tetroxide, triethylsilane, boron trifluoride etherate, and boron tribromide. These factors collectively contribute to significant ecological and practical challenges.^169,170^ To overcome these limitations, a biobased route toward **L-4** is highly desirable, and a first example has been developed by Kong et al. by engineering a fungal strain of *Aureobasidium melanogenum* to produce **L-4** in gram scale yield in a glucose fed-batch, posing a promising alternative in green chemistry over conventional chemical synthesis of **L-4** and related hydrazines.^80^

As an illustration, this biotechnologically produced **L-4** could, for example, be used as a building block in the synthesis of the angiotensin-converting enzyme inhibitor (ACE inhibitor) cilazapril (**9**). Further applications for KtzT homologues could arise from the evolution of the enzyme toward nonamino acid substrates. One potential target could be the pyridazine subunit in herbicide fluthiacet-methyl.^171^

#### 4.1.2 Linear Hydrazines as a Key Intermediate in NP Biosynthesis

##### 4.1.2.1 Intermolecular Hydrazine Formation via ATP-Dependent Hydrazine Synthetases

**Scheme 5:**
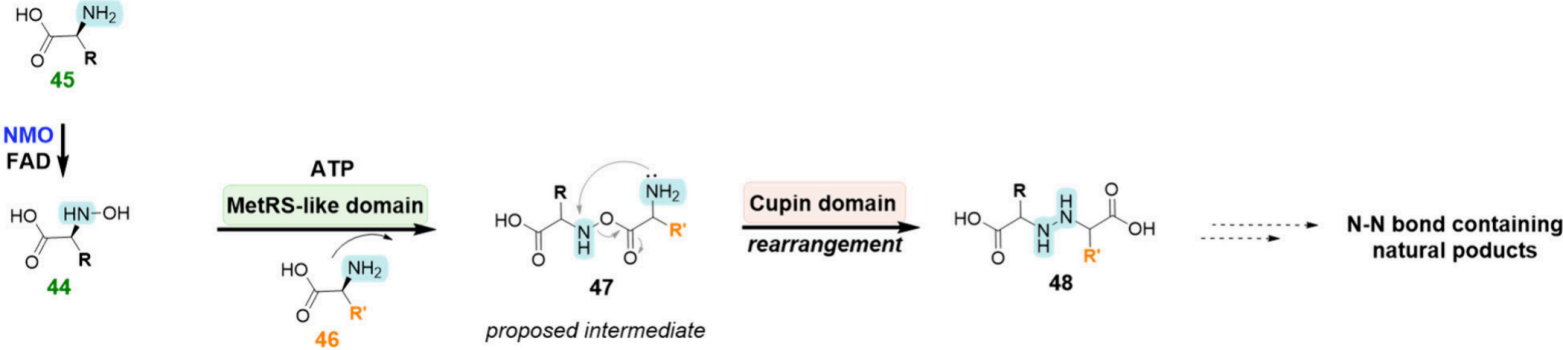
Proposed Reaction Mechanism of Hydrazine Synthetases^48^

The linear hydrazine functional group is employed in the biosynthesis of a wide range of NPs, following a common reaction mechanism.^25^ Recently, a review covering the members of the hydrazine synthetase family was published by Matsuda et al. that enlightens this new enzyme family in more detail.^48^ Hydrazine synthetases have recently gained attention for biocatalytic applications largely due to increasing the amount of available information regarding the mechanism of N–N bond formation and their reported substrate promiscuity. These didomain enzymes employ a methionyl-tRNA-synthetase-like (MetRS) domain and a cupin domain to catalyze the formation of an intermolecular hydrazine bond between amino acids [glycine (**39**), l-alanine (**40**), l-serine (**41**), l-glutamic acid (**42**), l-tyrosine (**43**)] and *N*^ω^-hydroxy-amino acids (**44**) in an ATP-dependent manner.^66,68,82,122−124^ As in the case of PZS, in order for the one nitrogen to be incorporated into the hydrazine bond, it must first be activated through *N*-hydroxylation of an amino acid (**45**) by a flavin-dependent NMO, often colocalized in their respective BGC.^82^ The catalytic mechanism of hydrazine synthetases is currently believed to involve three main steps (Scheme 5). First, a nonhydroxylated amino acid (**46**) is *O*-adenylated in an ATP-dependent manner by the MetRS-like domain to yield an aminoacyl adenylate. Second, a nucleophilic attack by the hydroxy group of *N*^ω^-hydroxy-amino acids (**44**) on the adenylated substrate is believed to yield an unstable intermediate. While this intermediate has not been isolated yet, the results of ^18^*O*-labeling experiments and LC-MS/MS analysis of the enzymatically formed species suggest that it may be an *O*-acylhydroxylamine ester intermediate (**47**).^81^ Subsequently, the cupin domain of the enzyme would catalyze an ester rearrangement and the formation of the intramolecular N–N bond, potentially via a nucleophilic attack of the primary amine on the secondary amine, releasing the hydrazine intermediate **48** that can be further incorporated in the biosynthesis of NPs.^66^

**Scheme 6:**
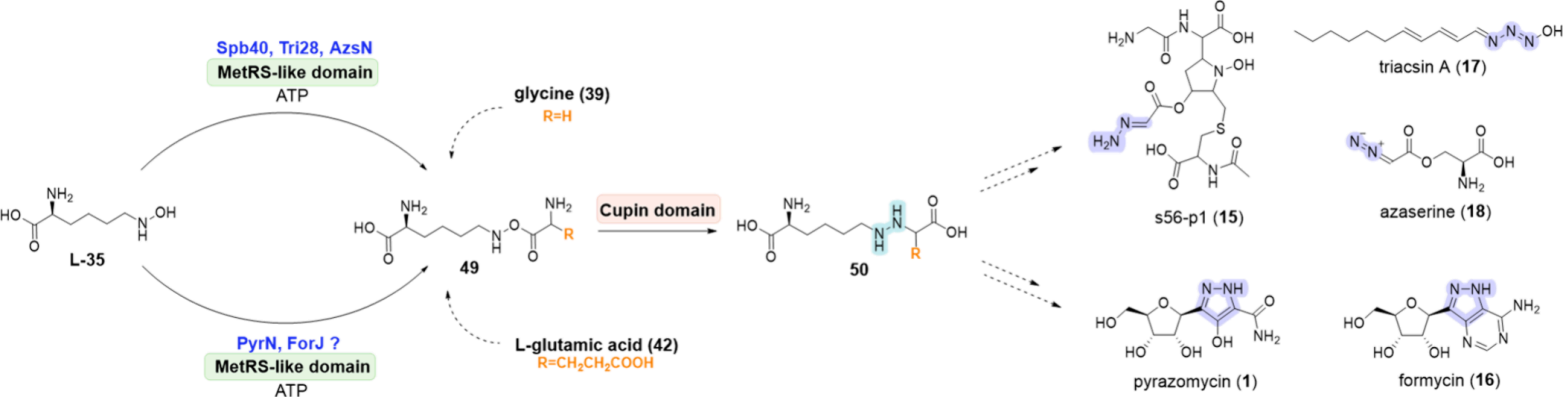
Biosynthesis of s56-p1 (**15**),^66^ Triacsin A (**17**),^68^ Azaserine (**18**),^124^ Pyrazomycin (**1**),^8^ and Formycin (**16**)^67^ NPs The biosynthesis of triacsins B-D, follow the same pathway as **17**.^178^ The N–N functional group directly formed by the NNzyme of interest is highlighted in blue, while other N–N bonds are highlighted in purple.

The elucidation of the biosynthetic pathway toward the NP s56-p1 (**15**), which is synthesized through a key hydrazine-containing intermediate,^48^ led to the discovery of the first zinc-dependent hydrazine synthetase, Spb40.^172^ In 2018, Matsuda et al. confirmed the *in vivo* activity of Spb40 from *Streptomyces* sp. SoC090715LN-17, which conjugates *N*^6^-hydroxy-l-lysine (**L-35**) and **39** to produce a putative ester intermediate **49** that likely rearranges into the hydrazine precursor *N*^6^-((carboxymethyl)amino)-l-lysine (**50**) (Scheme 6).^66^ The *N*-hydroxylation of l-lysine is catalyzed by the FAD-dependent monooxygenase Spb38 (UniProt ID: A0A1L7NQE9), a homologue of KtzI. The crucial hydrazine intermediate **50** is then subjected to oxidative cleavage by the FAD-dependent D-amino acid oxidase homologue Spb39, forming hydrazinoacetic acid (HAA, **51**), which is subsequently converted into the final NP **15** through still uncharacterized steps.^25^ The synthesis of **51** appears in many bacterial species^25^ and is an intermediate in the biosynthesis of other NPs, such as azaserine (**18**)^123^ and the triacsins (**17**).^68^

The biosynthesis of **17** (see also Section 4.4) also involves the formation of the hydrazine intermediate **50** catalyzed by the hydrazine synthetase Tri28 from *Kitasatospora aureofaciens* ATCC 31442.^68^ This intermediate is then further modified to the triazene functional group that is eventually found in the final NPs such as triacsins (**17**) (Scheme 6).^68^ Homologues of Spb40 and Tri28 have also been identified in the biosynthesis of **18** (Scheme 6), which involves the participation of hydrazine synthetases such as AzaE/Aza12 from *Glycomyces harbinensis* ATCC 43155^122,123^ or SFaza12/AzsN from *Streptomyces fragilis*.^123,124^ Although the enzymes responsible for the biosynthesis of the diazo moiety of azaserine (**18**) are not identified yet, similar to **17** it is synthesized from HAA, presumably via the oxidation of the hydrazine moiety, thereby providing the first example of a nitrous acid-independent diazo group biosynthetic pathway. In addition, structural studies have shown that these enzymes, unlike Spb40 and Tri28, for example, possess an additional C-terminal carrier protein domain that is structurally homologous to the PCP domain of carboxylic acid reductases,^122,173^ potentially allowing it to function as a carrier for the 2-hydrazineylideneacetyl (HDA or HYAA) intermediate.^124^ Hydrazine synthetases also play a crucial role in the biosynthesis of pyrazomycin (**1**), an antibiotic *C*-nucleoside featuring a pyrazole ring. Pyrazomycin **1** is produced by *Streptomyces candidus* NRRL 3601 via the *pyr* or *prf* BGC.^8^ Within the *pyr* BGC, the two-domain zinc-dependent enzyme PyrN, also known as PrfJ,^174^ catalyzes N–N bond formation in the hydrazine intermediate **50** formed between **L-35** and **42** (Scheme 6).^8^ The group of Du, conducted QM/MM calculations of the cupin mediated rearrangement of the intermediate **50**, in a PyrN homologue, named RHS1. This computational approach highlighted a key residue E69, in this specific domain, which plays a crucial role in the intramolecular rearrangement leading to the N–N bond formation.^81^ The general hydrazine synthetase mechanism is speculated to be also followed by the PyrN homologue ForJ in the biosynthesis of formycins (**16**), purine-related NPs with antibiotic, antiviral and antitumor activities.^16,175,176^ ForJ is proposed to catalyze the formation of an ester intermediate **49** between **L-35** and **42** followed by a rearrangement leading to the formation of a hydrazine intermediate **50** (Scheme 6).^177^ In contrast to PyrN, whose native function has been biochemically characterized, the activity of ForJ toward **L-35** and **L-42** is still under investigation. Nevertheless, a recent study has shown that ForJ is likely a hydrazine synthetase, due to the ability of its cupin domain to accept the native substrate of VlmO in the biosynthesis of **2** (see Section 4.1.2.3) forming the desired hydrazine intermediate **52** (Scheme 9).^67,174,177^

**Scheme 7:**
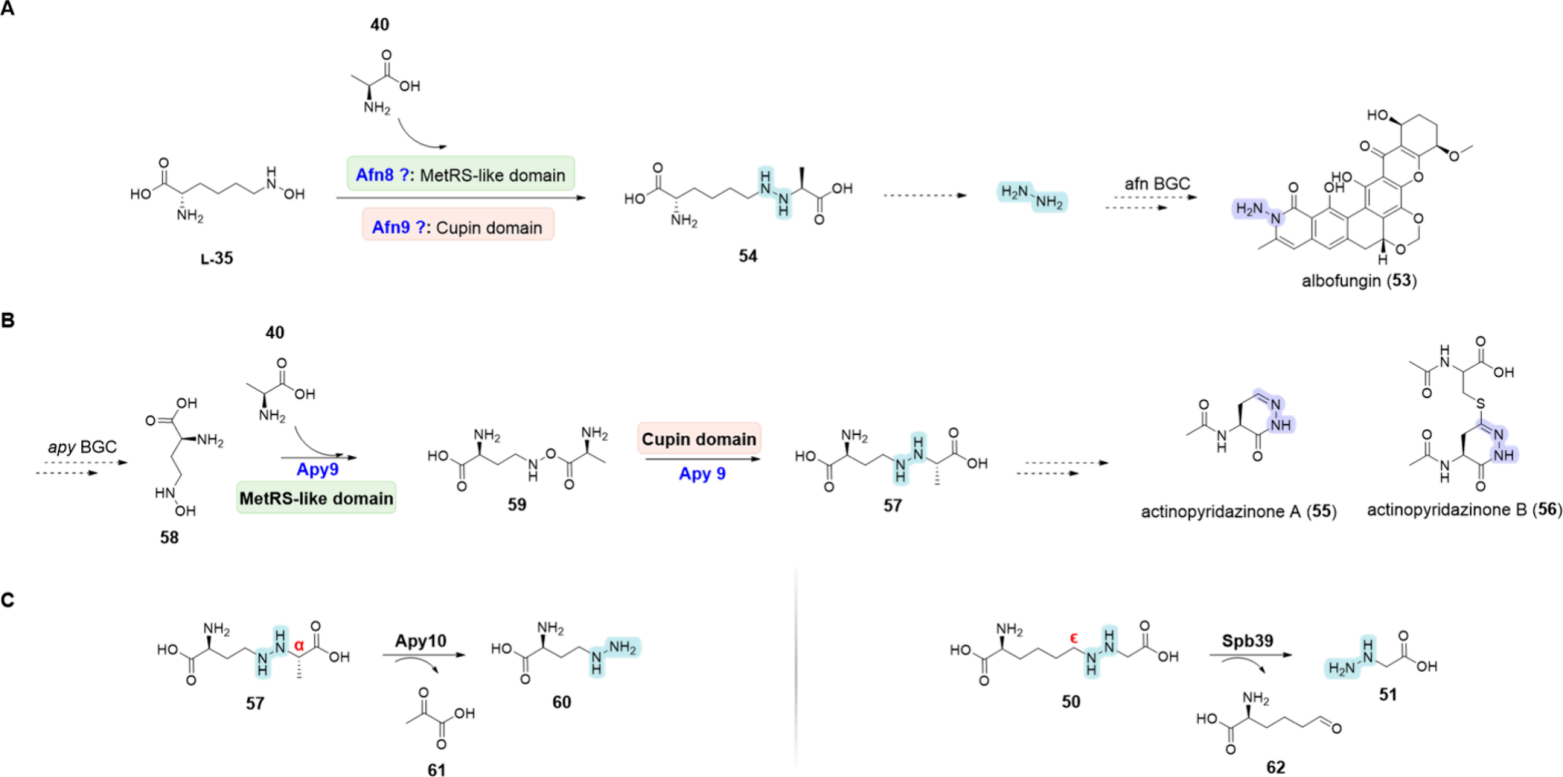
Biosyntheses of Albofungin, Triacsins B–D, and Actinopyridazinones and Regiospecific Cleavage of the Hydrazine Intermediate **A**, Biosynthesis of albofungin (**53**).^179^ The biosynthesis of triacsins B–D (**17-B**,**C**,**D**), follow the same pathway as triacsin A (**17-A**).^178^**B**, Biosynthesis of actinopyridazinones (**55**, **56**).^125^**C**, Regiospecific cleavage of the hydrazine intermediate (**57**, **50**) in the case of Apy10 and Spb39.^180^ The N–N functional group directly formed by the NNzyme of interest is highlighted in blue, while other N–N bonds are highlighted in purple.

Recently, the coordinated action of a putative hydrazine synthetase enzyme cascade and a hydrazine transferase for the N–N bond installation has been also reported in the biosynthesis of the antibiotic albofungin (**53**) (Scheme 7A).^179^ This discovery was made through the study of the corresponding BGC, *afn*, in *Streptomyces monomycini* CGMCC 4.3581 (DSM 41801). Analysis of this BGC revealed the presence of putative genes encoding a MetRS-like enzyme (Afn8), cupin (Afn9) and *N*^6^-lysine hydroxylase (Afn18). This enzymatic activity is reminiscent of linking two amino acid substrates as seen also in the biosynthesis of **1**, **15**, **16**, **17** and **18**. The authors hypothesized that Afn8, Afn9, and Afn18 are involved in an enzymatic cascade yielding a hydrazine intermediate (**54**) that is further converted to a free hydrazine molecule. They also elucidated that the asparagine synthase-like enzyme, Afn14 can catalyze the condensation of an aromatic polyketide precursor with this free hydrazine molecule, which is the first report of a hydrazine transferase activity in N–N bond formation pathways.^179^

Another interesting hydrazine synthetase identified by Matsuda et al. is Apy9, which was found in the BGC of actinopyridazinone A (**55**) and B (**56**) from *Streptomyces sp*. MSD090630SC-05.^125^ In this biosynthesis, the key enzyme Apy9 was shown to catalyze the formation of the hydrazine intermediate (**57**) via conjugation of *N*^4^-hydroxy-l-diaminobutyric acid (*N*^4^-hydroxy-l-DABA) (**58**) and l-alanine (**40**) into a DABA-Ala ester intermediate (**59**) (Scheme 7B). The hydroxylation of l-DABA to **58** is catalyzed by the NMO Apy11 (NCBI ID: BDC79917). In contrast to *N*-hydroxylases from other BGCs, such as Spb38, Tri26, ForK or PyrM, which are coupled to hydrazine synthetases active toward l-lysine, Apy11 showed no activity with either l-lysine or **L-27** as a substitute for l-DABA. Apy9 shows a broader substrate scope with respect to the hydroxylamine substrates, accepting **58** as well as **L-35** and **L-28**, but was restricted to **40** as the second amino acid substrate.^125^

In analogy to the biosynthesis of **15**, the hydrazine intermediate DABA-Ala (**57**) is oxidatively cleaved to l-2-amino-4-hydrazineylbutanoic acid (l-AHBA, **60**) by the FAD-dependent oxidoreductase Apy10 (NCBI ID: WP_057724699.1),^180^ which belongs to the same enzyme family as Spb39 and Tri27.^66,68^ However, Apy10 catalyzes the oxidation of a Cα–N bond to pyruvate (**61**), generating a γ-amine-based hydrazine (**60**), instead of the Cϵ–N bond to generate an α-amine-based hydrazine (such as **51**), releasing l-2-aminoadipate-6-semialdehyde (**62**) (Scheme 7C). This observation highlights the importance of regiospecific cleavage of the hydrazine intermediate between related pathways.^180^ In addition, Matsuda et al. identified a set of novel *N*-hydroxylases and hydrazine synthetases through SSN analysis in the genome of *Pseudomonas orientalis* DSM 17489. The *por* BGC encodes the *N*-hydroxylase Por9 (NCBI ID: WP_057724698.1) and the hydrazine synthetase Por11. *In vitro* characterization revealed the specificity of Por9 toward **L-28**, while *in vivo* Por11 was found to catalyze the formation of a hydrazine bond between **58** or **L-28** and **39**.^125^

As described above, hydrazine synthetases are frequently involved in the construction of the hydrazine functional group, which serves as a key intermediate for the construction of the final NP,^81^ and are widely distributed across a large number of organisms. Both Zhao et al. in 2021 and Matsuda et al. in 2024 conducted phylogenetic analyses and enzyme mining that revealed the hydrazine synthetases to be part of a bigger and diverse family of biocatalysts. (Table 2).^81,82^

**Table 2:** List of Other Identified Hydrazine Synthetases

**Hydrazine synthetase**	**Organism**	**NCBI ID**	**ref**
D5UDN8-D5UDN9	*Cellulomonas flavigena* DSM 20109	ADG76495/ADG76494	(81)
A0A552E3D4	*Microcystis aeruginosa* Ma_SC_T_19800800_S464	TRU28978	
A0A126Y2P7	*Streptomyces albidoflavus*	AMM09270	
A0A3S9PCD5-A0A3S9PCD2	*Streptomyces luteoverticillatus*	AZQ70060/AZQ70059	
A0A0L0QKX8-A0A0L0QQM7	*Virgibacillus pantothenticus*	KNE19216/KNE20884	
A0A423LFS6	*Pseudomonas fluorescens*	RON67156	
A0A291T5 V8-A0A291T5Z9	*Streptomyces malaysiensis*	QDL68185/ATL88544	
A0A2B9TI29	*Bacillus cereus*	PGO71607	
Q8KGM6	*Mesorhizobium japonicum* R7A	CAD31310	
Dpn5-Dpn6	*Streptomyces luteoverticillatus* CGMCC 15060	WP_126912625	(82)
Bac1-Bac2	*Bacillus paranthracis* AM04S-42	WP_076855484	
Col1-Col2	*Colwellia* sp.	MBL4898055	
Kit1-Kit2	*Kitasatospora gansuensis* DSM44786	WP_184911086	
Cor1-Cor2	*Corallococcus llansteffanensis* CA051B	WP_120641657	
Ral1-Ral2	*Ralstonia solanacearum* CFBP2957	WP_013209038	

**Figure 4:**
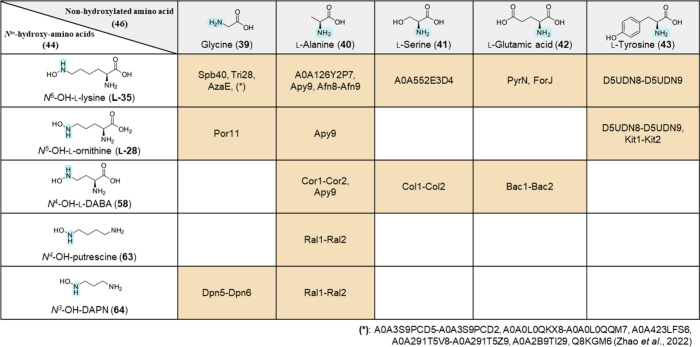
Current scope of conjugates formed by respective MetRS/cupin hydrazine synthetases.^8,66−68,81,82,125^

Furthermore, an analysis of the amino acid specificity within the hydrazine synthetase enzyme family revealed a wide substrate selectivity for nonhydroxylated amino acids (**46**) and *N*^ω^-hydroxy-amino acids (**44**), which are essential for the construction of the N–N bond (Figure 4). In addition to that, bioinformatic studies focusing on the nonhydroxylated amino acid binding pockets of the MetRS-like domains revealed eight key amino acid residues essential for substrate specificity and thus represent potential enzyme engineering targets for the targeted engineering of biocatalysts catalyzing intermolecular N–N bond formation.^82^ In this sense, the biocatalytic activity of hydrazine synthetases to form aliphatic hydrazine subunits based on *N*-hydroxylated substrates and an amine-containing substrate has the potential to replace hazardous alkylated hydrazine derivatives commonly used to introduce this substructure, such as in the synthesis of the anti-ischemic drug meldonium or the chemotherapeutic agent procarbazine (**10**). However, the substrate scope for the *N*-hydroxylated substrates must be further extended beyond α-amino acids, as has already been shown for substrates *N*^4^-OH-putrescine (**63**) and *N*^3^-OH-DAPN (**64**). Unfortunately, the nonhydroxylated amine-containing substrates are mechanistically limited to amino acids and therefore further transformations such as decarboxylation reactions may be required in the development of biocatalytic synthesis routes.

##### 4.1.2.2 Intermolecular Hydrazine Formation via Putative Nitrite-Dependent Hydrazine Synthases

**Scheme 8:**
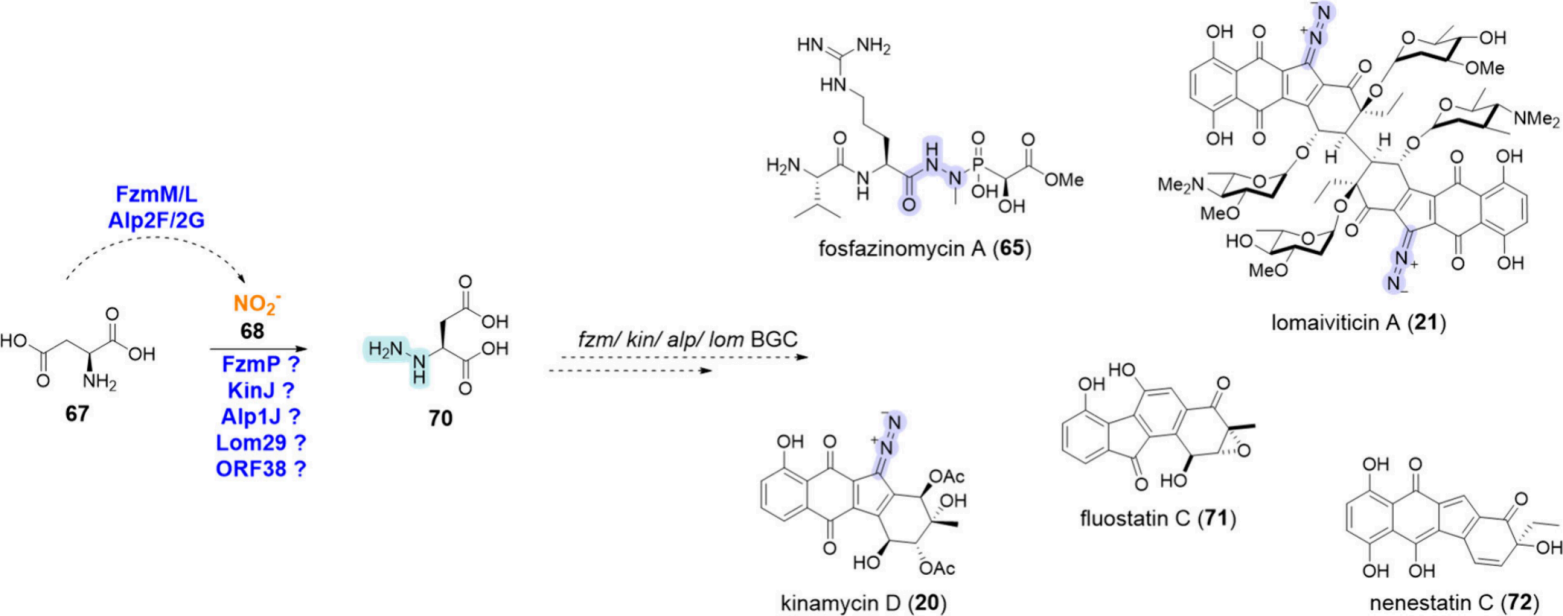
Biosynthesis of Kinamycin D (**20**),^126,182^ Lomaiviticin A (**21**),^126,128^ Fosfazinomycin A (**65**),^126^ Fluostatin C (**71**),^129^ and Nenestatin C (**72**)^182^ via Putative Nitrite-Dependent Hydrazine Synthases The N–N functional group directly formed by the NNzyme of interest is highlighted in blue, while other N–N bonds are highlighted in purple.

Fosfazinomycin (**65**) is an N–N bond-containing NP with a distinctive phosphonohydrazide moiety (Scheme 8). Efforts to explore its BGC and identify the NNzyme involved (organism *Streptomyces* sp. XY332) revealed a flavin-dependent oxygenase, FzmM (UniProt ID: A0A0N0UQ79). In the initial step of the biosynthesis, FzmM catalyzes the oxidation of l-aspartic acid (**67**) to *N*-hydroxy-l-aspartic acid. The hydroxylated species then acts as a substrate for the enzyme FzmL (UniProt ID: U5YN81), to produce nitrite (**68**).^181^ These enzymes are homologous of CreE and CreD, the first identified enzymes responsible for nitrite liberation from **67** in cremeomycin BGC, as explained in detail in Section 4.2. Labeling experiments in the native producing organism of **65**, *Streptomyces* sp. NRRL S-149,^126^ confirmed the incorporation of nitrous acid (**69**) into the corresponding N–N bond. This N–N bond formation was assigned to a hypothetical protein, FzmP, that is proposed to construct a hydrazinosuccinic acid intermediate **70** from **67** (Scheme 8). However, further biochemical characterization needs to confirm the native activity of this enzyme.^126^

Hydrazinosuccinic acid (**70**) not only plays a role in the biosynthesis of **65** but has been proposed to also serve as key intermediate in the NPs kinamycins (**20**), lomaiviticins (**21**), fluostatin C (**71**) and nenestatin C (**72**). Following comparison studies in the whole genome of the producer organisms of those NPs, it became apparent that despite their structural differences with **65**, they share a set of homologous genes. Specifically, in the genome of *Streptomyces murayamaensis* ATCC 21414, producer of **20**, a homologue of FzmP called KinJ is proposed to facilitate the N–N bond formation.^45,126^ A very recent study on the biosynthesis of the same family of NPs showed that the final diazo group is installed after the backbone of the NP has been constructed by a protein called AlpH (PDB ID: 8H3T).^182^ AlpH is an *O*-methyltransferase-like enzyme that introduces the entire l-glutamylhydrazine intermediate (gluN_2_H_3_) into the backbone of **20**. The origin of the N–N bond in the intermediate gluN_2_H_3_ is proposed to be generated by the putative FzmP homologue, Alp1J. It is noteworthy that in the same *alp* BGC, the proteins Alp2F and Alp2G, also homologues of FzmM and FzmL, were identified.^127^ In the biosynthesis of lomaiviticin (**21**), another hypothetical NNzyme was assigned due to its shared homology with FzmP, called Lom29.^128,129,182^ Although a diazo N–N bond is not always found in the final NPs as in the case of **71** and **72**, their biosynthetic pathway will include a step of diazo incorporation.^129,183,184^ In the biosynthesis of **71**, an uncharacterized enzyme has been proposed to facilitate this step, identified as ORF38 (Scheme 8).^129^ Despite the efforts to explore the BGCs of the aforementioned NPs, all putative enzymes catalyzing **70** formation remain to be biochemically characterized and further investigation is required to confirm their native function in these different biosynthetic pathways. Nevertheless, the biocatalytic potential of such NNzymes would be very useful, as it would allow the introduction of a terminal hydrazine from an amine and nitrite.

##### 4.1.2.3 Azoxy-Containing NP Biosynthesis via Hydrazine Intermediates

**Scheme 9:**
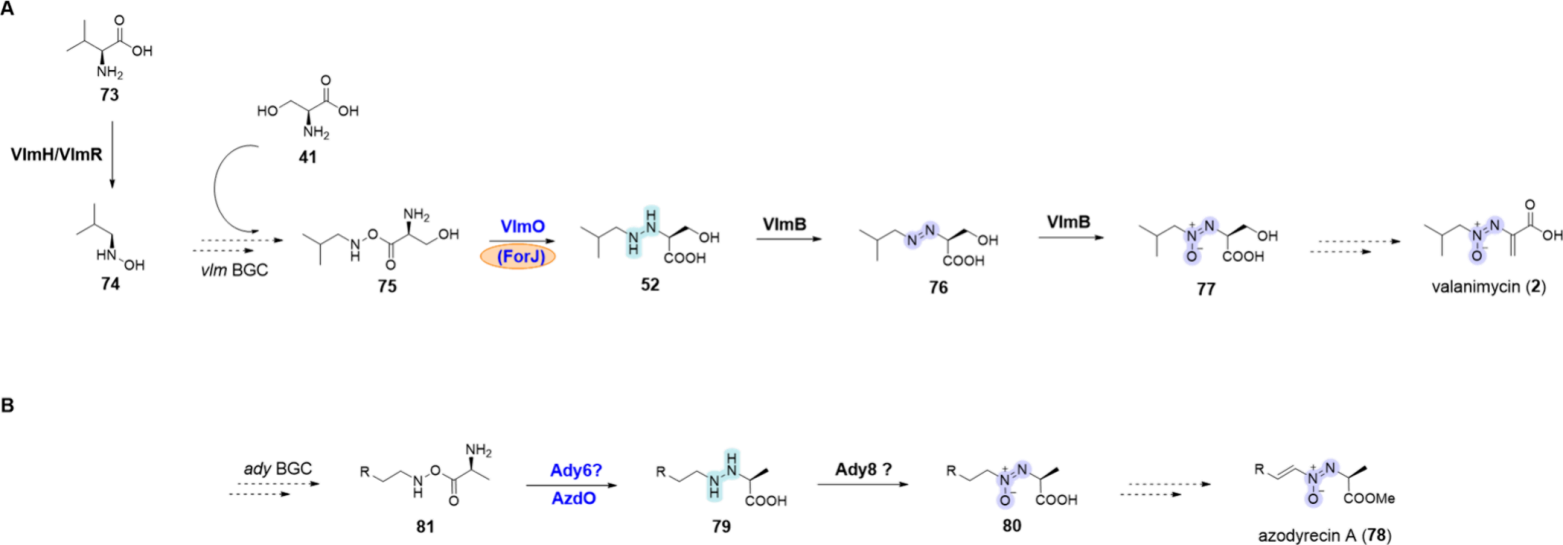
Biosynthesis of Azoxy-Containing NPs via Hydrazine Intermediates **A**, Biosynthesis of valanimycin (**2**).^130^**B**, Biosynthesis of azodyrecin A (**78**).^132^ The N–N functional group directly formed by the NNzyme of interest is highlighted in blue, while other N–N bonds are highlighted in purple.

VlmO is a unique example of membrane-bound NNzyme that is responsible for the synthesis of the hydrazine intermediate (**52**) in the valanimycin (**2**) biosynthetic pathway. The formation of an N–N bond via VlmO is chemically analogous to the reactions catalyzed by zinc-dependent hydrazine synthetases from the cytosol cupin family (e.g., ForJ), yet exhibits no homology with them.^81,130,185,186^ Despite the fact that the enzymatic basis responsible for azoxy bond formation has remained largely enigmatic, early research on the biosynthesis of **2** indicated that l-valine (**73**) and l-serine (**41**) undergo a hydrazine-azo-azoxy pathway via a *N*-isobutylhydroxylamine intermediate (**74**).^186^ This hypothesis was confirmed when it was found that the hydroxylation step can be catalyzed by a two-component, flavin-dependent monooxygenase (VlmH,VlmR).^9,187−190^ In the BGC of **2**, VlmA (NCBI WP_014134059.1) catalyzes the condensation of **74** with seryl-tRNA to form an unstable ester intermediate, *O*-seryl-isobutylhydroxylamine (**75**).^67,131^ Additionally, the heme-like diiron-dependent oxygenase VlmB (UniProt ID: E4N6B3) was shown to be an essential in the biosynthesis of **2**, accepting **52** formed by VlmO and converting it to the final azoxy-containing NP, via an azo- (**76**) and an azoxy- containing intermediate (**77**) (Scheme 9A).^130^

Iron binding in VlmO is likely mediated by four essential residues (D51, H82, H110, and D114) as site-directed mutagenesis experiments revealed.^130^ These residues are located in a potential solvent-accessible cavity found in a structure model of VlmO that was predicted by AlphaFold. Other recently found homologues of the NNzyme VlmO and the oxygenase VlmB have been identified in the biosynthesis of many azoxy-containing NPs, such as KA57-A (KaO-KaB (NCBI ID: BBC93013.1)), elaiomycins (ElaO-ElaB (NCBI ID: WP_114244573.1)) and azodyrecins (**78**) (AzdO-AzdB (BGC ID: BGC0002805)).^130^

*In vivo* studies for the characterization of the enzymatic pairs ElaO/ElaB and KaO/KaB revealed that conserved steps must be involved in the biosynthesis of aliphatic azoxy metabolites. In addition to that, VlmO/VlmB and their homologues share flexible substrate specificity, while it was proven that they can accept substrates with various aliphatic chains. In azodyrecin (**78**) biosynthesis, the VlmO homologue AzdO catalyzes an intramolecular N–N bond formation producing a hydrazine product (**79**) that is further transformed into the azoxy-containing precursor (**80**) of the final NP (Scheme 8B).^131^ In addition, the membrane proteins Ady6 and Ady8 (GenBank ID: BDI55415.1/BDI55432.1) of the ferritin-like superfamily from *Streptomyces* sp. RM72 (LC712332) and *Streptomyces* sp. A1C6 (LC712331) were proposed as the enzyme pair catalyzing the ester (**81**) rearrangement to the hydrazine intermediate (**79**) and subsequently to the azoxy-containing molecule (**80**) (Scheme 9B). The biochemical characterization of these enzymes is still pending, but the homology with VlmO supports the hypothesis that Ady6 may act as a hydrazine synthase.^132^ The biocatalytic activity of VlmO/VlmB and their homologues toward aliphatic azoxy compounds is promising, as the hydroxylamine substrate is not limited to amino acids. However, as with MetRS/cupin hydrazine synthetases, the nonhydroxylated amine substrate is mechanistically restricted to amino acids. To date, several biologically active NPs have been identified, including calvatinin, azoxybacillin, and elaiomycin, which possess antifungal or antibacterial activities.^83,191^ The intriguing chemical structures of azoxy compounds and their diverse biological activities have prompted research in the field of natural product chemistry, total synthesis, and biochemistry to identify new routes toward novel azoxy compounds.

### 4.2 Diazo Functional Group

**Scheme 10:**
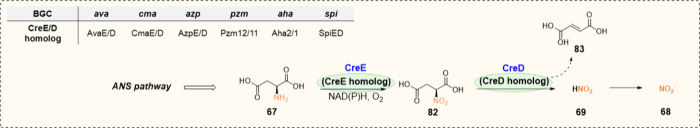
Formation of Nitrite (**68**) through the ANS Pathway^192^

One of the main questions arising in the enzymatic synthesis of diazo moieties in NPs is the origin of the nitrogen donor. Exploring the biosynthesis of cremeomycin (**3**), Sugai et al. identified nitrous acid (**69**) as the source of the distal nitrogen in the diazo group in **3**. Nitrous acid is synthesized via an enzymatic pathway, later named as the aspartate-nitrosuccinate (ANS) pathway, that involves two key enzymes: the FAD-dependent monooxygenase CreE (UniProt ID: A0A0K2JL70), which catalyzes the iterative oxidation of **67** to nitrosuccinic acid (**82**), and CreD (UniProt ID: A0A0K2JL82), which converts **82** to nitrous acid (**69**), releasing fumaric acid (**83**) (Scheme 10).^192^ Based on the observations made by Winter et al., who established nitrite (**68**) as the nitrogen source for the N–N bond in azamerone (**84**) biosynthesis,^193^ Sugai et al. investigated the genome of the azamerone producer *Streptomyces* sp. CNQ-525. Notably, they found *creE* and *creD* homologues forming an operon at a different locus from the putative azamerone BGC. Given the high potential of actinobacteria to produce secondary metabolites, they analyzed additional actinobacterial genomes and found the ANS pathway widely distributed, often near genes for secondary metabolite biosynthesis. They focused their research on examining known BGCs of NPs containing N–N bonds, such as the hydrazine-containing **65**, and identified CreE and CreD homologues, named FzmM and FzmL, respectively, as described in Section 4.1.2.2. This suggests that nitrous acid (**69**) could serve as the nitrogen donor not only in diazo-containing compounds like **3** but also in other N–N bond-containing NPs, such as **65** and **84**.^192^ Further research confirmed the presence of CreE and CreD homologues in multiple BGCs as described in Table 3 that also contains putative or confirmed diazo-forming enzymes.^136,138^

**Table 3:** CreE and CreD Homologs Found in BGCs Related to Diazo-Group Formation

**Natural Product**	**BGC**	**CreE Homologue (Accession Number)**	**CreD Homologue (Accession Number)**	**ref**
alazopeptin (**86**)	azp	AzpE (NCBI ID: WP_035850953.1)	AzpD (NCBI ID: WP_035850955.1)	(134)
tasikamides A–C (**88**–**90**)	aha	Aha2 (NCBI ID: UMM61380.1)	Aha1 (NCBI ID: UMM61379.1)	(135)
spinamycin (**93**)	spi	SpiED (NCBI ID: WP_189300634.1)		(136)
penzoemycins A and B (**98**, **99**)	pzm	Pzm12 (NCBI ID: UUJ74624.1)	Pzm11 (NCBI ID: UUJ74623.1)	(137)
avenalumic acid (**95**)	ava	AvaE (NCBI ID: BDI54816.1)	AvaD (NCBI ID: BDI54817.1)	(138)
*p*-coumaric acid (**104**)	cma	CmaE (whole genome GenBank ID: CP007155.1)	CmaD (whole genome GenBank ID: CP007155.1)	(139)

**Scheme 11:**
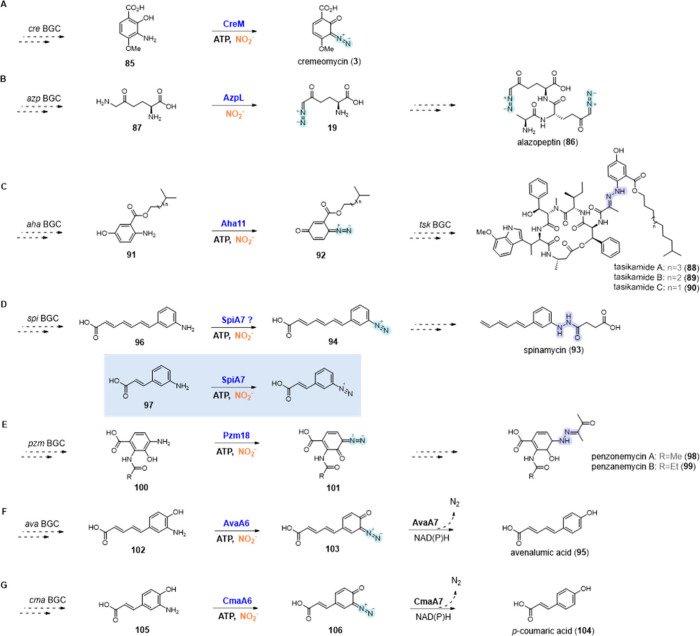
Proposed Biosynthetic Pathways Involving Diazo-Bond-Forming NNzymes **A**, Biosynthesis of cremeomycin (**3**).^133^**B**, Biosynthesis of alazopeptin (**86**).^134^**C**, Biosynthesis of tasikamides A-C (**88**–**90**).^135^**D**, Biosynthesis of spinamycin (**93**).^136^**E**, Biosynthesis of penzonemycins A–B (**98**, **99**).^137^**F**, Biosynthesis of avenalumic acid (**95**).^138^**G**, Biosynthesis of *p*-coumaric acid (**104**).^139^ The N–N functional group directly formed by the NNzyme of interest is highlighted in blue, while other N–N bonds are highlighted in purple.

Once the origin of the nitrogen was established, the next question to address was whether these diazo moieties were formed enzymatically and which enzymes were responsible for this process. The first diazo group-forming enzyme discovered was CreM, an enzyme part of the cremeomycin BGC (*cre)* found by Waldman et al. in the genome of *Streptomyces cremeus* NRRL 3241.^10^ Cremeomycin (**3**) is a photosensitive *o*-diazoquinone with antibacterial and antiproliferative activity that was isolated for the first time in 1967.^194,195^ CreM, predicted to be a fatty acid-CoA ligase of the acyl-CoA ligases, nonribosomal peptide synthetases and luciferases (ANL) superfamily, catalyzes the diazotization of 3-amino-2-hydroxy-4-methoxybenzoic acid (3,2,4-AHMBA) (**85**) with **68** to produce **3** both *in vivo* and *in vitro* (Scheme 11A). Initial characterization of N–N bond-forming activity of CreM was challenging due to the spontaneous formation of the diazo group in **3** under certain culture conditions, as observed by the authors when testing different culture media, some of which promoted this unintended reaction.^133^ To address this issue, the *creM* gene was expressed and the biosynthesis of **3** reconstituted in *Escherichia coli*, whose media did not catalyze nonenzymatic diazotization. However, the low production levels and chemical instability of **3** hindered its detection *in vivo*. To further confirm the catalytic activity of CreM, the authors introduced a mutation into a highly conserved residue, E352, which is known to coordinate an essential Mg^2+^ ion required for ATP binding in members of the fatty acid-CoA ligase family. This mutation resulted in the complete abolishment of **3** production.^133^

Another diazo-group containing molecule is alazopeptin (**86**), a NP synthesized by *Streptacidiphilus griseoplanus* and *Kitasatospora azatic*a.^134,196,197^**86** is a tripeptide comprising two molecules of the diazo-containing amino acid DON (**19**) and one molecule of l-alanine (**40**).^198,199^ The antibiotic and antitumor properties of **86** are of interest from a pharmaceutical perspective.^197^ In 2021, Kawai et al. revealed the complete biosynthetic pathway of this compound (*azp* BGC) and identified the NNzyme of this pathway as the transmembrane protein AzpL, which uses **68** and 5-oxo-l-lysine (**87**) toward the formation of the diazo intermediate **19**, which is incorporated twice into the final product (Scheme 11B). The following step in this pathway is the production of *N*-Ac-DON, from a *N*-acetyltransferase protein AzpI (NCBI ID: WP_035850924.1). Kawai et al. conducted a comparative analysis of potential AzpL homologues and identified a number of conserved tyrosine, serine and glutamate residues that are likely to be involved in the catalytic mechanism.^134^ Furthermore, an alanine screening was conducted, which revealed that the mutation Y93A completely abolished the production of *N*-Ac-DON. Conversely, the substitution of the same residue with phenylalanine resulted in a reduction in the formation of *N*-Ac-DON. These findings indicate that Y93 plays a pivotal role in the catalytic mechanism of AzpL.^134^ Further efforts have been made to characterize other enzymes of the biosynthetic pathway.^200^

Diazo groups are not always found in the final NP, instead, they frequently occur in intermediates that are subsequently transformed into other N–N bond-containing functional groups, such as hydrazones. This is the case with tasikamides A-C (**88**–**90**), compounds that have a hydrazone group linking the cyclic peptide backbone to an alkyl 5-hydroxylanthranilate (AHA, **91**) moiety. They were isolated from *Streptomyces tasikensis* P46 by Ma *et a*l for the first time in 2022.^135^ They identified two different BGCs responsible for the biosynthesis of this molecule. The first, *tsk* BGC, encodes a nonribosomal peptide synthetase (NRPS) pathway for assembling the cyclic pentapeptide scaffold and the second BGC, *aha*, encodes the genes in charge of synthesizing the alkyl AHA moiety (**91**). This cluster encodes genes that share sequence similarity to genes from the biosynthetic pathway of **3**. Aha11 (CreM homologue) is an ATP-dependent arylamine-diazotizing (AAD) enzyme that performs the diazotization reaction of **91**, forming the intermediate diazo-AHA (**92**). Then, the diazo compound undergoes a nonenzymatic Japp–Klingemann reaction that couples it with the cyclic peptide generating the hydrazone-containing tasikamides (**88**–**90**) (Scheme 11C).^135^ The same research group deleted the *aha11* gene and obtained three new tasikamides (I, J and K) that show different structure to **88**-**90**, in which the 2 subunits are connected by an enaminone bridge instead of the hydrazone moiety. This demonstrates the implication of Aha11 in the enzymatic assembly of this junction, confirming its role as an NNzyme.^201^

It is plausible that the same biosynthetic logic employed for the construction of the N–N bond of spinamycin (**93**), an antifungal antibiotic discovered in 1966 that contains a hydrazide moiety.^202,203^ Kawai et al. isolated this NP from *Streptomyces albospinus* JCM3399.^136^ It was demonstrated that a diazo intermediate (**94**) plays a pivotal role in the incorporation of the hydrazine bond into the final product (Scheme 11D). By querying ANS pathway genes (Scheme 10), the spinamycin biosynthetic gene (*spi*) cluster was uncovered, which surprisingly contained CreE/CreD homologues in the form of a natural fused protein called SpiED (NCBI ID: WP_189300634.1).^136^ In terms of its structural composition, **93** exhibits an aryl polyene moiety, which is similar to that observed in avenalumic acid (**95**) (Scheme 10F). The latter was the subject of a previous study by the same research group, and another NNzyme called AvaA6 was identified in the corresponding BGC.^138^ While both BGCs display similarities, they also exhibit differences that reflect the structural variations observed in the final NPs. An ATP-dependent homologue of the diazotase AvaA6 was identified within the *spi* cluster and named SpiA7. It is hypothesized that this enzyme performs diazotization of the aromatic amine 6-(3-aminophenyl)-2,4,6-heptatrienoic acid **96**, utilizing nitrite **68** produced by SpiED as a nitrogen source. This results in the formation of unstable diazonium intermediate **94**, that rapidly decomposes into cinnamic acid, due to the lack of stabilizing hydroxy group in the ortho position. The activity of SpiA7 toward this substrate was postulated on the basis of *in vitro* evidence being inconclusive. However, its activity toward 3-aminocinnamic acid (**97**) provided a confirmation that SpiA7 is a diazonium-forming NNzyme. It is noteworthy that SpiA7 is active toward anilines lacking a hydroxyl group. This is particularly significant given that other diazo NNzymes require the presence of a hydroxyl group for diazotization. For example, in the biosynthesis of avenalumic acid (**95**), **88**–**90** and **3**, the aromatic substrates of diazotization enzymes possess a hydroxyl group at the para or ortho position of the amino group to be diazotized. Following the diazotization, the tautomerization of the hydroxyl group contributes to the stabilization of the diazo intermediate. However, this stabilizing mechanism is absent in the synthesis of **93**, which results in the low stability of the diazo intermediate and its subsequent rapid transformation within the biosynthetic pathway. This transformation may occur spontaneously via the Japp-Klingemann reaction, or it could be enzyme-mediated, although the responsible enzyme has not yet been identified.^136^ The authors employed Japp-Klingemann chemically to be able to detect the product formed in the *in vitro* assays.^136^

A similar mechanism of hydrazone moiety construction was observed in the biosynthesis of **98** and **99**. Recently isolated from the marine organism *Streptomyces* sp. SCSIO 40020, these novel molecules possess a hydrazone moiety and a 3-hydroxyanthranilic acid (3-HAA) core. After isolation, Liu et al. identified the putative gene cluster encoding the enzymatic machinery for its biosynthesis (*pzm* BGC). The 3-HAA core was proposed to be derived from a chorismate pathway involving the genes *pzm6* to *pzm9* (NCBI BGC: ON345781.1). This cluster also contains a CreM homologue identified as the AMP-binding protein Pzm18, that is proposed to incorporate nitrate **68** with a putative substituted benzoic acid intermediate (**100**), leading to the formation of the diazo moiety in **101**. Finally, the nonenzymatic Japp-Klingemann coupling reaction is predicted to construct the hydrazone moiety (Scheme 11E). Further experimental data are required to confirm the activity of all the enzymes involved in this biosynthetic pathway.^137^

Until now, the diazo NNzymes described in the preceding paragraphs have been shown to create an N–N bond that either remains in the final molecule as a diazo moiety or undergoes further transformation to e.g. hydrazone or pyridazine. However, this principle is not universally applicable. In certain NPs, diazo intermediates are formed initially, but then undergo a deamination process, resulting in the elimination of the N–N bond as nitrogen gas (N_2_). This is the case for avenalumic acid (**95**), a phenolic acid originally found in oat plants, where it occurs as a structural motif in avenanthramide compounds.^204^ This NP was later isolated from the bacterium *Rhodococcus* sp. RV157.^205^ In their search for novel enzyme chemistries that exploit **69** derived from the ANS pathway similarly to CreM, Kawai and colleagues mined the genomes and identified the *ava* cluster in *Streptomyces* sp. RI-77.^138^ Despite their efforts, they were unable to isolate **95** from this organism, likely because it is a dormant secondary metabolite BGC under the given culture conditions. Therefore, they expressed this cluster heterologously in *Streptomyces albidoflavus*, successfully demonstrating the production of the compound. During the biosynthesis of **95**, an ATP-dependent diazotase homologue to CreM, AvaA6, performs the diazotization of an aromatic amino group. Specifically, the enzyme performs diazotization of 3-aminoavenalumic acid (3-AAA) (**102**) into 3-diazoavenalumic acid (3-DAA) (**103**). This is followed by the reductive enzymatic substitution of the diazo group with a hydride, liberating N_2_, carried out by the enzyme AvaA7 (NCBI ID: BDI54815.1) (Scheme 11F). The authors further performed a genome mining which revealed that more than 100 actinobacteria carry BGCs similar to the *ava* cluster, indicating that this NP or its derivatives may be produced by a wide variety of actinobacteria.^138^

Another NP without an N–N bond in its structure but with a diazo intermediate is *p*-coumaric acid (**104**). Its structure is similar to **95**, differing only in the length of the carbon chain. It is a precursor in the flavonoid biosynthetic pathway, normally derived from phenylalanine or tyrosine (**43**).^206^ Kawai et al. employed the *ava* BGC as a query to identify the *cma* BGC in the genome of the rare actinomycete *Kutzneria albida* JCM 3240.^139^ Heterologous expression of the cluster and *in vitro* enzyme assays demonstrated its role in the biosynthesis of **104**. In this *ava*-like BGC, an ATP-dependent diazotase homologue to AvaA6 was found, named CmaA6. This enzyme catalyzes the diazotization of 3-aminocoumaric acid (3-ACA, **105**), forming the intermediate 3-diazocoumaric acid (3-DCA, **106**). It is noteworthy that CmaA6 exhibited diazotization activity with 3-AAA (**102**), the substrate of AvaA6, with significantly higher efficiency than AvaA6. Following diazotization, analogous to the biosynthesis of **95**, **106** undergoes a denitrification catalyzed by CmaA7, releasing N_2_ and forming **104** (Scheme 11G). The reason behind the evolution of such a specialized biosynthetic pathway in the secondary metabolism of actinomycetes, which includes diazotization and denitrification, remains unclear. This pathway is utilized to synthesize a common metabolite, such as **104**, despite the lack of a clear selective advantage.^139^

**Scheme 12:**
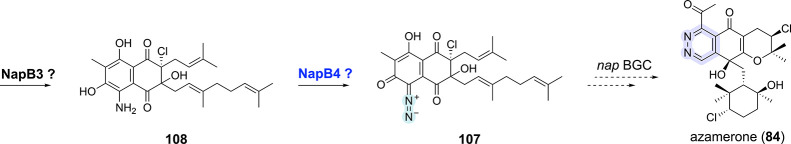
Biosynthesis of Azamerone (**84**) via the *nap* BGC^193,207^^,^ The N–N functional group directly formed by the NNzyme of interest is highlighted in blue, while other N–N bonds are highlighted in purple.

Diazo groups can also be further transformed into pyridazine motifs, as seen in the azamerone (**84**) biosynthesis. This NP is a pyridazine-containing compound isolated from the marine *Streptomyces* sp. CNQ-766, which belongs to the napyradiomycin class of NPs.^207^ Winter et al. hypothesized that the BGC responsible for the biosynthesis of **84** must be similar to the napyradiomycin BGC (*nap*), previously identified in *Streptomyces* sp. CNQ-525 and *Streptomyces aculeolatus* NRRL 18442.^193,208^ This group also conducted feeding studies, which suggested the potential involvement of **49** as a nitrogen source for the distal nitrogen atom in the diazo-containing precursor of **84**, designated as SF2415A1 (**107**).^193^ This hypothesis was further supported by the discovery of CreE and CreD homologues forming an operon at a different locus from the putative azamerone BGC.^192^ This discovery implies that the diazo moiety observed in the intermediate stages of the biosynthesis of **84** could potentially be formed through enzymatic processes. Winter and colleagues have hypothesized that the putative aminotransferase NapB3 (NCBI ID: ABS50480.1) may facilitate the transfer of an amino group to the aromatic ring of SF2415B1 (**108**), thereby introducing the initial nitrogen atom required for subsequent N–N bond formation. Subsequently, an unknown NNzyme can transfer another nitrogen atom to form the diazo group-containing precursor **107**.^25,193^ Later on, Waldman et al. identified a CreM homologue within the BGC of **84** that could potentially be this elusive NNzyme. To further investigate this, we conducted a BLAST search querying CreM in the genome of *nap* BGC-containing organisms *Streptomyces aculeolatus* and *Streptomyces* sp. CNQ-525. This search uncovered the CreM homologues (43% identity) named NapB4 in both organisms (Scheme 12). Regarding the pyridazine ring present in the structure of **84**, it has been postulated that this ring is formed via an oxidative rearrangement of a diazo intermediate.^193,207^

In general, given the reactivity of diazo groups, they are typically proposed as elusive reaction intermediates and in organic synthesis, as chemical probes for the modification of proteins and nucleic acids, and as building blocks in the biosynthesis of pharmaceutically relevant compounds. The potential of NNzymes to create reactive diazo intermediates in synthetic applications was recently demonstrated *in vitro* for CmaA6. This enzyme formed the diazo group that then was used to generate phenyldiazene derivatives via C–N bond formation.^209^ Apart from the use of diazo compounds as reactive intermediates, the presence of diazo groups in NPs, such as **20** and **21**, confers upon these molecules the ability to intercalate DNA, thereby rendering them promising candidates for anticancer therapies.

### 4.3 *N*-Nitroso Functional Group

#### 4.3.1 Nitrosamide (*N*-Nitrosourea) Functional Group

**Scheme 13:**
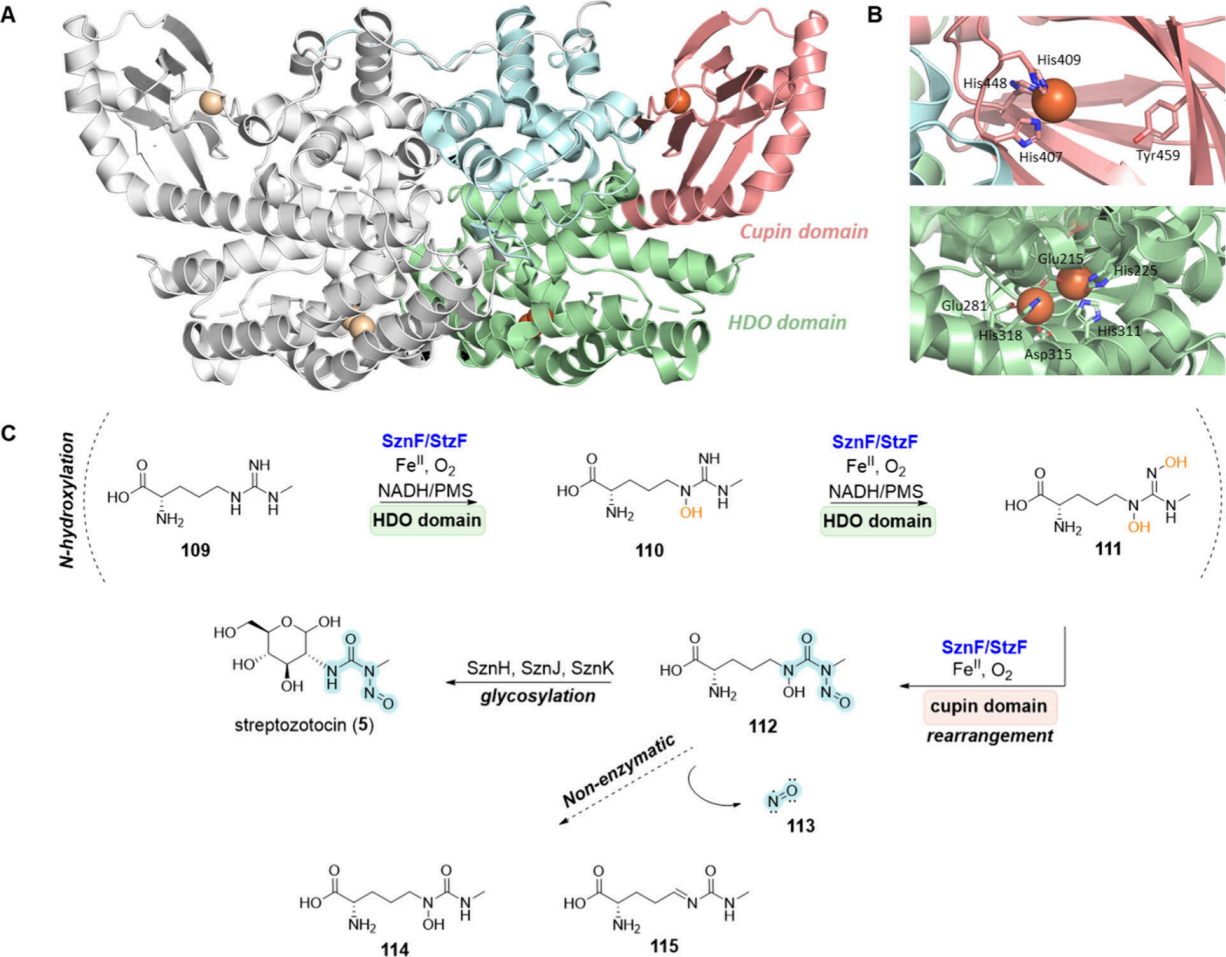
SznF/StzF, Essential Residues for Activity, and SznF/StzF Mechanism in the Biosynthesis of Streptozocin **A**, Crystal structure of SznF/StzF (PDB ID: 6VZY), represented as homodimeric protein.^215^ The different domains are depicted with distinguished colors. In blue, the *N*-terminal domain is depicted, in green the HDO central domain and in red the *C*-terminal cupin domain. The orange spheres represent the iron atoms in the active sites. **B**, Essential residues for activity H407, H409, H448 and Tyr 459 in the cupin domain (top) and E215, H225, E281, H311, D315, and H318 in the multinuclear central domain (bottom). **C**, Proposed mechanism of SznF/StzF in the biosynthesis of streptozocin (**5**).^140,215^ The N–N functional group directly formed by the NNzyme of interest is highlighted in blue.

Streptozocin (**5**) (streptozotocin, or trade name Zanosar) is a *N*-nitrosourea-containing NP that was first isolated in the late 1950s from *Streptomyces achromegenes* subsp. *streptozoticus* and documented as a new antibiotic.^140,210,211^ Nowadays, the commercial formulation is used as an antineoplastic drug to treat pancreatic cancer.^13,212^ Although **5** has been in use for more than half a century, its BGC was not identified until 2019 by the Balskus and Ryan groups. Balskus’s group sequenced and mined the genome of *Streptomyces achromegenes* subsp. *streptozoticus* NRRL 2697 and found the *szn* BGC that encodes the NNzyme SznF (UniProt ID: A0A411MR89, Scheme 13 A and B).^140^ The group of Ryan identified the responsible NNzyme, named in this study StzF, in the genome of *Streptomyces achromegenes* subsp. *streptozoticus* NRRL 3125, and named the BGC *stz*.^116^ After further investigation of the cluster and *in vitro* characterization of SznF/StzF it was found that this NNzyme acts synergistically with an arginine-guanidino methyltransferase, SznE/StzE (UniProt ID: A0A411EW25), to accept *N*^ω^-methyl-l-arginine (**109**) as a native substrate. SznF/StzF hydroxylates sequentially both of the unmethylated nitrogen atoms of the guanidine group, forming two hydroxyl intermediates, *N*^δ^-hydroxy-*N*^ω^-methyl-l-arginine (**110**) and *N*^δ^-hydroxy-*N*^ω^-hydroxy-*N*^ω^-methyl-l-arginine (**111**). This dihydroxylated intermediate undergoes a rearrangement resulting in the production of a *N*-nitrosourea intermediate (**112**), which is then converted further to **5** via glycosylation, likely mediated by the enzymes SznH, SznJ, and SznK. The intermediate **112** can also undergo a nonenzymatic degradation, producing nitric oxide (**113**) and the degradation products **114** and **115** (Scheme 13C).^116,140^ It is noteworthy that SznF/StzF is the only NNzyme that has been identified to construct the *N*-nitrosourea functional group. Although it was previously speculated that the *N*-nitroso group originated from the intramolecular incorporation of **68**, similar to all known *in vivo N*-nitrosations,^213,214^ feeding experiments demonstrated that *Streptomyces achromogenes* subsp. *streptozoticus* incorporates the intact guanidine group of **109** into the *N*-nitrosourea product **112** without utilizing ^15^N-labeled nitrite, nitrate, or ammonium salts to generate the *N*-nitroso moiety.^140^ Considering all of the above-mentioned, SznF/StzF exhibits a unique enzymatic activity, performing both hydroxylation and N–N bond formation through an intramolecular rearrangement reaction without requiring a coupled *N*-hydroxylase or the use of **68**. In light of the observation that **112** is formed in the biosynthetic pathway of **5**, it can be hypothesized that the intermediates formed by SznF/StzF may act as donors for the N-nitrosourea subunit used in chemotherapeutic drugs such as carmustine (**11**) or lomustine (**12**). In addition to that, SznF/StzF comprises one of the NNzymes with a resolved crystal structure (PDB IDs: 6M9R, 6XCV, 6VZY, 6M9S).^215^ The X-ray crystallography revealed that this NNzyme is a homodimer composed of three domains: an N-terminal domain, responsible for intramolecular interactions during dimerization, a heme-oxygenase–like diiron oxidase and oxygenase (HDO) central domain, which catalyzes two consecutive *N*-hydroxylations and a C-terminal monoiron cupin domain, which catalyzes the final rearrangement and the formation of the N–N bond (Scheme 13A).^140,215^ The enzyme is known to possess two active sites, one in each iron-containing domain. Substitution of any of the metal-binding residues (E215, H225, E281, H311, D315, or H318) in the multinuclear central domain with alanine resulted in abolition of SznF/StzF activity (Scheme 13B). Similarly, substitutions of residues H407, H409 and H448 in the cupin domain led to the accumulation of the dihydroxylated intermediate **111**, without any product formation.^140^ In addition, computational analyses and mechanistic studies conducted by Chen’s group on the HDO and the cupin domain of SznF/StzF provide a basis for further exploration of this biocatalyst.^216,217^ Mechanistic analysis of the HDO central domain activity and the hydroxylation process revealed that the rate-limiting step in the formation of the monohydroxylated intermediate **110** is a hydroxyl rebound, whereas for the second hydroxylation and the formation of intermediate **111**, it is a hydrogen abstraction.^216^ Additionally, analysis of the cupin-mediated rearrangement indicated that the residue Tyr459 facilitates a proton transfer essential for this rearrangement step. These findings highlight the critical role of these residues in the enzyme’s catalytic activity.^217^

#### 4.3.2 Nitrosohydroxylamine Functional Group

**Scheme 14:**
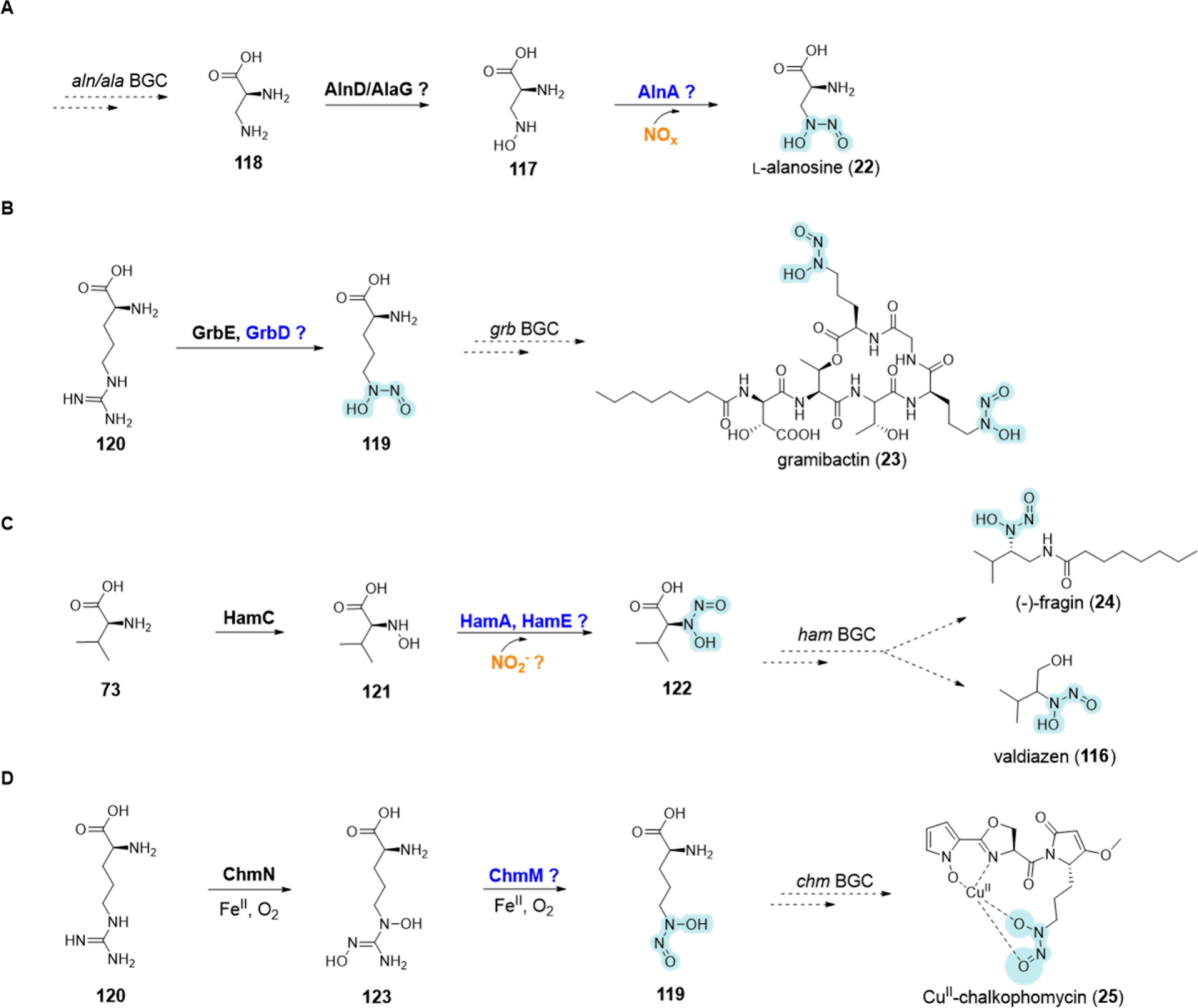
Biosynthesis of NPs with a Nitrosohydroxylamine Functional Group **A**, Biosynthesis of l-alanosine (**22**).^141^**B**, Biosynthesis of gramibactin (**23**).^25,144^**C**, Biosynthesis of (−)-fragin (**24**) and valdiazen (**116**).^146^**D**, Biosynthesis of Cu^II^-chalkophomycin (**25**).^103^.

In the biosynthesis of several NPs, including l-alanosine (**22**), gramibactin (**23**), (−)-fragin (**24**), valdiazen (**116**) and chalkophomycin (**25**), specific genes have been identified that encode biocatalysts with the potential to function as NNzymes. These enzymes are hypothesized to catalyze the formation of a nitrosohydroxylamine group, which is subsequently incorporated into the final structure of the respective NPs.^25,146,218^ Although the activity of these biocatalysts remains under investigation, the structural similarity they share with SznF/StzF^116^ supports the hypothesis that they may indeed function as NNzyme in each biosynthetic pathway.

l-Alanosine (**22**), a noncanonical amino acid with antiviral and antitumor properties, was originally isolated in 1966 from *Streptomyces alanosinicus* ATCC 15710.^219^ The BGC responsible for the biosynthesis of **22** was recently identified and designated as *ala*(142) and *aln*.^141^ The *aln* BGC includes the enzyme Aln *A*, a putative NNzyme featuring a cupin domain and an AraC-like DNA-binding domain. Aln *A* is hypothesized to act as either a transcriptional regulator or to catalyze N–N bond formation via the cupin active site, possibly targeting *N*^3^-hydroxy-l-diaminopropionic acid (**117**) to form the nitrosohydroxylamine group in **22**. The hydroxylation of l-diaminopropionate (**118**) is hypothesized to be catalyzed by a putative flavin-dependent acyl-CoA dehydrogenase, AlaD/Aln *G* (UniProt ID: A0A6H1Z5U0/A0A6B9JBY3) (Scheme 14A). The origin of the distal *N* in **22** remains under debate due to conflicting isotope feeding studies suggesting either the ANS pathway involving CreD and CreE homologues (AlaJ/Aln *N* and AlaI/Aln *M*) (UniProt ID: A0A6B9JDZ4 and A0A6H1Z626) or NO_*x*_ species produced by nitrate-nitrite reductases as possible sources.^141,142^ This latter hypothesis is further supported by recent discoveries, as newly identified *aln* BGCs in other *Streptomyces* species lacking ANS pathway genes suggest that the nitrate and nitrite reductases present may provide the distal nitrogen necessary for N–N bond formation.^218^

Similarly, GrbD, which possesses a HDO and a C-terminal cupin domain, is likely responsible for catalyzing the N–N bond formation in the biosynthesis of **23** using hydroxy-arginine as a substrate (Scheme 14B).^143^ Gramibactin (**23**) was isolated in 2018 from *Paraburkholderia graminis* and contains l-graminine (**119**) moiety which is used as precursor to construct the final NP.^144^ GrbE (UniProt ID: B1G5G8), sharing homology with known arginine hydroxylases such as AglA/AlpD,^220,221^ Mhr24,^220−222^ and DcsA,^220,222,223^ is speculated to hydroxylate **120** to produce the precursor **119**.^143^ The authors also hypothesized that the N–N bond is formed between N^δ^ and N^ω^ of the guanidinium group of **120**,^143^ in contrast to previous studies that identified l-ornithine as the precursor.^25^ The role of GrbE in **120** hydroxylation and GrbD in the oxidative rearrangement of hydroxy-arginine in the formation of the N–N bond in **119** is still under research. Interestingly, GrbD and GrbE have been used as queries to identify novel BGCs responsible for the biosynthesis of other graminine-containing siderophores, such as tistrellabactins A and B, which feature GrbD and GrbE homologues in their BGCs.^224^ The same approach led to the discovery of other nitrosohydroxylamine-containing compounds like gramibactin B, megapolibactin, plantaribactin and gladiobactin.^145^

(−)-Fragin (**24**), another nitrosohydroxylamine-containing NP with antifungal and antibacterial activity,^146^ is likely also constructed by the involvement of an *N*-oxygenase (HamC, UniProt ID: B4EHM6) that hydroxylates l-valine **73** to **121**, as well as a HDO protein, HamA, and a polyketide cyclase or dehydratase, HamE, that are hypothesized to facilitate N–N bond formation, yielding the key nitrosohydroxylamine precursor **122**. HamB, a cupin domain protein, might also play a role in the biosynthesis of **24**. In addition, the BGC also encodes genes that are involved in valdiazen’s (**116**) production, a molecule similar to **24** (Scheme 14C). However, further studies are needed to confirm these roles and also the source of the distal nitrogen, which since now is hypothesized to derive from NO_2_^–^.^25,146^

Another NP whose biosynthesis follows hydroxylation of an amino acid precursor catalyzed by an *N*-oxygenase with subsequent nitrosohydroxylamine formation catalyzed by a SznF/StzF homologue is Cu^II^-chalkophomycin (**25**).^103^ Its potential applications include neurodegenerative disease treatment and Cu^II^-based antitumor therapeutics.^225^ In 2024, the Balskus group characterized **25** from *Streptomyces anulatus* ATCC 11523 and elucidated its BGC.^226^ ChmM and ChmN (UniProt ID: A0A7H0NKC5) are key enzymes that have been studied for their potential involvement in the formation of the diazeniumdiolate ion. ChmN is a heme-dependent guanidine *N*-oxygenase that converts **120** to dihydroxyguanidine (**123**). The SznF/StzF homologue ChmM, possesses an HDO domain but lacks the occupancy of all the conserved amino acids, and a C-terminal cupin domain that is hypothesized to catalyze the subsequent rearrangement of **123** to the final N–N bond of **119**. This intermediate is likely further converted to **25** (Scheme 14D). The homology of ChmM and ChmN with GrbD and GrbE strengthens the hypothesis that the intermediate of **119** is involved in its biosynthesis.^103^ However, *in vitro* studies of ChmM against free hydroxyarginine derivatives have not confirmed its activity, requiring further biochemical characterization in the future.^226^ Although the nitrosohydroxylamine group is currently of limited synthetic interest, its chelating properties could prove a valuable asset in the development of biocatalytically available protein inhibitors that could have potential in drug discovery.

### 4.4 Triazene Functional Group

**Scheme 15:**
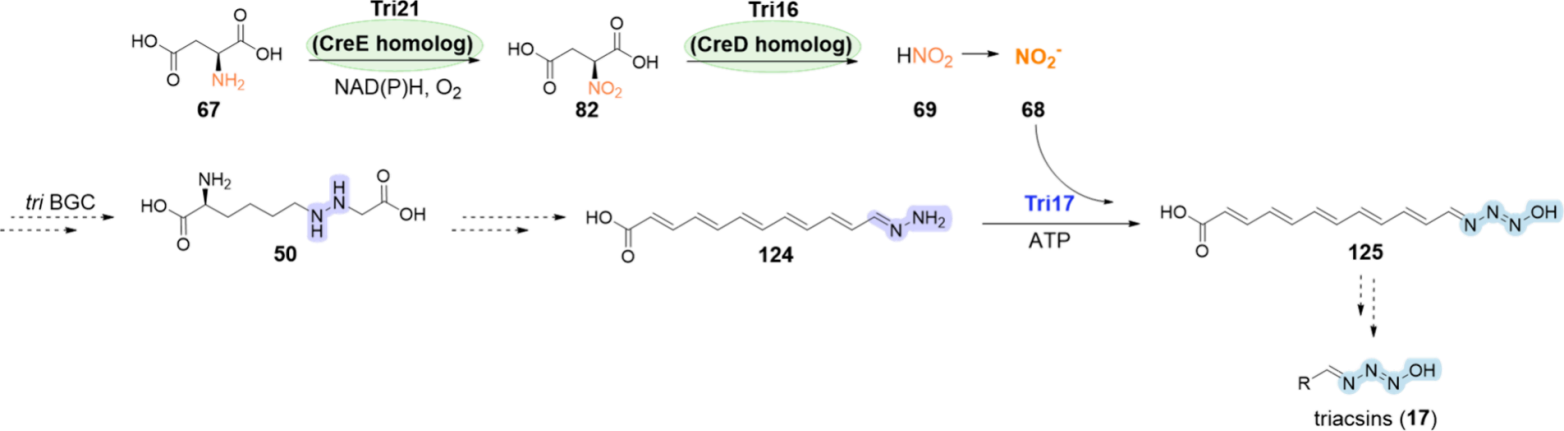
Biosynthesis of Triacsins (**17**) via the *tri* BGC^68^ The N–N functional group directly formed by the NNzyme of interest is highlighted in blue, while other N–N bonds are highlighted in purple.

The only known group of NPs containing an *N*-hydroxytriazene moiety are the triacsins (**17**), which represent a distinctive functional group with pronounced acyl-CoA synthetase inhibitor properties.^68^ In 2018, Twigg et al. discovered the BGC responsible for the biosynthesis of **17** in *Streptomyces tsukubensis* NRRL 18488.^178^ Later, in 2021, Del Rio Flores et al. determined that the enzymes Tri28 (Spb40 homologue, 75% sequence similarity) and Tri17 (CreM homologue, 40% sequence similarity) of *Kitasatospora aureofaciens* ATCC 31442 are responsible for the formation of the first and second N–N bonds, respectively, in the biosynthesis of **17**.^68^ The authors also identified ANS’ pathway homologues Tri21 and Tri16, within the *tri* BGC and demonstrated their role in generating the **68** that serves as the donor of the third nitrogen atom.^68^ As mentioned in Section 4.1.2.1, the hydrazine intermediate **27** in the biosynthesis of **17** is constructed by the hydrazine synthetase Tri28. In the later steps of this pathway, the hydrazine moiety **50** is transformed to a hydrazone (**124**) that acts as a substrate for the NNzyme Tri17. This enzyme catalyzes an ATP-dependent conjugation of **68** and the hydrazone intermediate **124** to generate a *N*-hydroxytriazene (**125**) that is further converted to the family of triacsins (**17**) (Scheme 15).^68^ Though not having been studied exhaustively yet, the proposed mechanism describes Tri17 first activating **68** by adenylation, allowing for nucleophilic attack by the distal hydrazone-nitrogen.^178^ Subsequently, tautomerization of the formed *N*-nitrohydrazide would yield the *N*-hydroxytriazene.^178^ Initially, Tri17 was shown to be specific regarding the hydrazone moiety and the acyl chain length, but less selective for different acyl chain modifications (e.g., converting undeca-2,4-dien-1-ylidenehydrazine to **17**),^68^ leading to its designation as a promiscuous *N*-nitrosylase. It was later shown by Del Rio Flores et al. that Tri17 is also capable of forming azides from alkylhydrazones, hydrazines, pyrrolidines, piperidines, arylamines and arylhydrazines, as well as diazo compounds from anilines, similar to Aha11 and CreM.^227^

In conclusion, Tri17 is a highly versatile enzyme that accepts a variety of functional groups and substrates, suggesting its potential for the biocatalytic synthesis of pharmaceutical compounds. The recent determination of the crystal structure of Tri17 (PDB ID: 9BQ0, 8TF7), has provided further insights into its substrate coordination and catalytic mechanism, thereby opening up the potential for further exploitation of its promiscuity.^227^

### 4.5 N–N Bond-Containing Aromatic Heterocycles

The mechanisms underlying the formation of heterocyclic N–N bonds in nature remain relatively underexplored, though they are currently the focus of ongoing research. The biosynthesis of these N–N bond-containing heterocycles commonly follows a pathway that involves a hydrazine intermediate. This strategy has been observed in the synthesis of nonaromatic heterocycles, such as actinopyridazinones (**55**,**56**)^125,180^ and aromatic heterocycles, like the pyrazole scaffold found in natural products such as pyrazomycin (**1**)^8^ and formycin (**16**)^67^ (see Section 4.1.2.1). Despite the absence of confirmed NNzymes capable of directly forming aromatic heterocycles such as pyrazoles, tetrazoles, pyridazines and so forth, potential NNzymes for triazole construction have recently been identified and will be discussed in greater detail herein.

#### 4.5.1 Triazole Functional Group

**Scheme 16:**
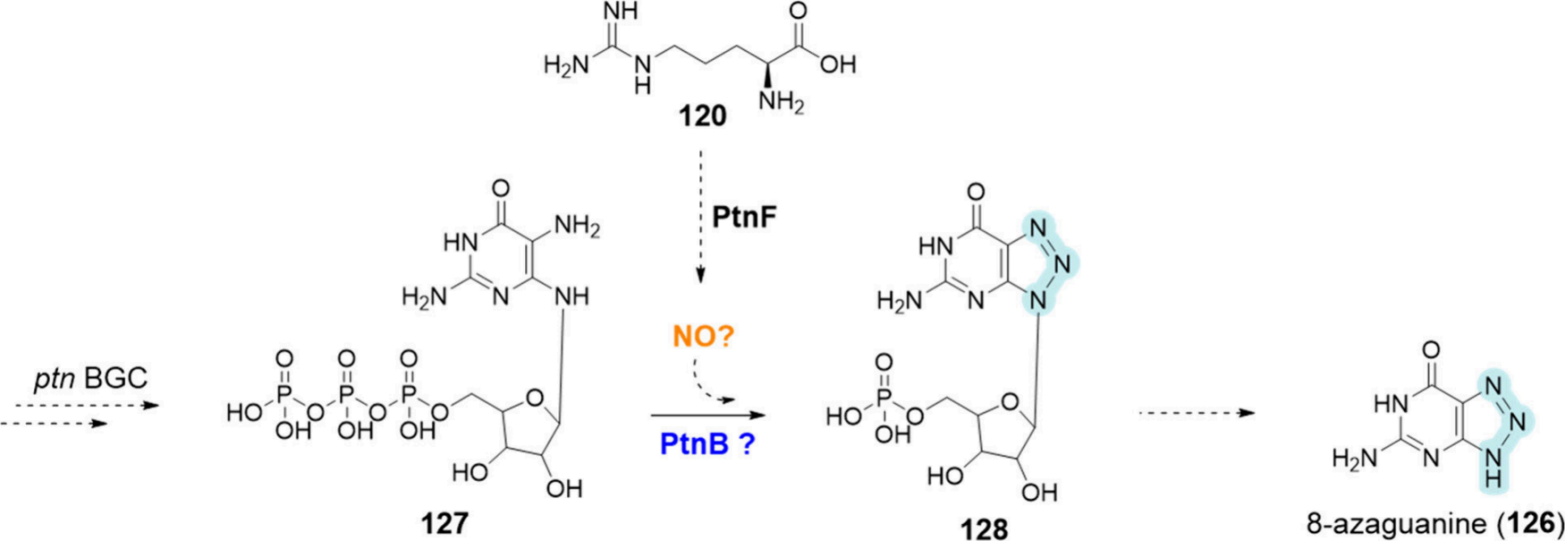
Proposed Biosynthesis of 8-Azaguanine (**126**)^147^

Currently, several putative NNzymes have been identified as potential candidates for directly catalyzing N–N bond formation in a triazole moiety. These enzymes (PtnB or 8-AzgE) are postulated to be responsible for the N–N bond formation in the biosynthesis of 8-azaguanine (**126**),^147,148^ also known as pathocidin, a compound that was originally reported as a synthetic guanine antagonist and subsequently isolated as a NP synthesized by *Streptomyces albus* subsp. *pathocidicus* (also known as *Streptomyces pathocidini*).^228,229^ This compound is notable for its structure as a guanine analogue, featuring a rare naturally occurring 1,2,3-triazole fused with a pyrimidine ring. As a purine analogue, **126** functions as an antimetabolite, displaying a wide array of biological activities, including anticancer, antiviral and antifungal properties.^228,230^ As previously mentioned, the most common precursors for N–N bond formation are hydroxylamines and nitrous acid. However, Zhao et al. revealed that nitrogen atom of the triazole moiety can also be provided by a bacterial nitric oxide synthase (NOS), named PtnF (UniProt ID: A0A6G9KI63), found in the 8-azaguanine (**126**) BGC *ptn*.^147^ NOS converts **120** to l-citrulline, releasing nitric oxide (**113**).^147^ Regarding the N–N bond construction, the authors suggested that a NO-derived reactive nitrogen species might be responsible for the assembly of the triazole moiety in a nonenzymatic fashion.^147^ But, despite demonstrating that this 1,2,3-triazolopyrimidine scaffold can be assembled nonenzymatically, the possibility of the existence of an NNzyme in the BGC was not excluded. It was proposed that PtnB, a small protein with no close homologues or predicted functional domains, may be the NNzyme, given its classification as an iron-binding metalloprotein.^147^ As seen in previous sections, metalloproteins have been linked to N–N bond formation in the biosynthesis of various compounds including streptozocin (**5**),^140^ s56-p1 (**15**),^66^ Piz (**L-4**)^76^ and pyrazomycin (**1**).^8^ It is speculated that PtnB may act toward **127** using NO, in order to construct the triazole moiety in molecule **128**, however, its activity has not yet been confirmed *in vitro*, necessitating further investigation to elucidate its role (Scheme 16).^147^ Hou et al. identified as well the BGC responsible for the biosynthesis of **126** in *Streptomyces pathocidini* (8-*azg* BGC) and agreed that a more efficient enzymatic pathway should exist for the synthesis of the triazole moiety and named this putative NNzyme 8-AzgE.^148^ This BGC has also been identified in other 8-azaguanine producing species like *Streptomyces morookaense* DSM 40503^231^ and *Streptomyces hoynatensis* KCTC 29069, although no 8-azaguanine-type product has been identified so far from this strain.^148^ Further studies are required to confirm the involvement of these clusters in the biosynthesis of **126**. In conclusion, the biocatalytic potential of triazole-forming enzymes may be considerable if further research can demonstrate a broader applicability to aromatic diamines in ortho position. This is particularly relevant given the role of triazoles as building blocks in drug discovery, as evidenced by their use in the nucleoside analog ticagrelor.^232^

## 5 Challenges and Opportunities for NNzymes

For a broad synthetic application of NNzymes, a large substrate scope including different carbon backbone structures and functionalizations would be ideal. In contrast, the substrate portfolio of most NNzymes presented in this review, if studied at all, indicate that only compounds closely related to the native substrates are accepted.^68,76,78,125,130,135,138^ For instance, in addition to AHA (**91**) (Scheme 11C), Aha11 also converts related esters of shorter methyl and butyl alcohol units, but not related compounds without a *para*-hydroxy group or 2-amino-5-hydroxybenzoic acid. Nevertheless, the natural substrate scope of enzymes involved in N–N bond formation can be extended by enzyme engineering, as has been shown for AzoC in azoxymycin biosynthesis,^89^ making enzymatic routes more attractive for synthetic applications. In this context, it is worth mentioning that two recent studies investigate nitroreductases and nonspecific peroxygenases (UPOs), respectively, for the generation of the reactive nitroso and hydroxylamine intermediates required for the spontaneous formation of azoxy groups.^90,233^ In addition, nitroreductases could also be used to produce azobenzene compounds under photocatalytic conditions. However, according to our definition, they cannot be classified as NNzymes, as the final N–N bond is formed spontaneously. Nevertheless, the potential of AzoC, nitroreductases and UPOs for the synthesis of azoxy groups is worth mentioning, as these enzymes are well characterized and readily applicable for synthetic applications compared to most NNzymes. To achieve the same level of applicability for NNzymes, they need to be engineered for a broader substrate scope and higher catalytic activity. However, in-depth structural and mechanistic knowledge of the enzymes is required for (semi)-rational engineering. Currently, only the crystal structure of the KtzT homologue PipS, Tri17, and SznF/StzF is available, which has made it possible to identify residues crucial for activity and to elucidate the reaction mechanism with which N–N bond formation is catalyzed.^79,140,215,227^ For other enzymes such as VlmO^130^ or PyrN,^82^ for which no crystal structures are available, AlphaFold models were created to identify the residues involved in catalysis. Elucidation of the residues involved in NNzyme activity is the first step to gain mechanistic insights that are crucial for rational engineering of enzymes toward new substrates. Therefore, AlphaFold can be a valuable tool to promote further mechanistic studies when no crystal structure is available. However, for some NNzymes such as FzmP, KinJ, PtnB or Ady6, where the homology to well-characterized enzymes is low or even the catalyzed reaction is uncertain,^45,126,132,147^ further structural and biochemical studies are required to gain mechanistic insights. The general lack of mechanistic information makes it difficult to expand the substrate range of NNzymes and could explain why for the KtzT homologue PAI2, none of the seven designed mutants could extend the substrate scope of the enzyme.^78^ In addition to enzyme engineering, the natural diversity of enzymes can also be used to access new substrates. For example, Matsuda et al. identified eight binding pocket residues in naturally occurring cupin/MetRS-like enzymes that specify their substrates as either Gly, Ala, Ser, Glu or Tyr. These enzymes can be used to synthesize various hydrazine intermediates **48** (Scheme 5),^82^ highlighting how enzyme mining could help expand the range of accessible NNzymes’ products.

Several pharmaceutically interesting N–N bond-containing products, such as *N*-nitrosamine derivatives,^89,119,132^ long-chain aliphatic *N*-hydroxytriazenes^68,178^ and α-hydrazino acids, are already accessible via NNzymes. However, other important substrate classes, such as N–N bond-containing aromatic heterocycles, azoxy compounds, linear azines and aliphatic diazo compounds are not yet accessible, although they occur in nature (see Figure 2).^147,234−236^ In addition to enzyme engineering, further biochemical characterization and identification of NNzymes could help to obtain the above-mentioned functional groups. To represent a real alternative for the chemical synthesis of N–N bonds, NNzymes must also have high activity, good soluble expression and stability in order to reduce the amount of enzyme required. However, kinetic studies have so far only been carried out for KtzT, the KtzT homologue PAI2, Tri17 and AvaA6.^68,76,78,138^ KtzT shows a *k*_*cat*_/*k*_*M*_ value of 57.5 s^–1^ mM^–1^, which is very promising for synthetic applications, while PAI2, Tri17 and AvaA6 with *k*_*cat*_/*k*_*M*_ values of 0.12, 2.25, and 0.03 s^–1^ mM^–1^, respectively, would need to be further improved, e.g. by enzyme engineering. Consequently, in addition to enzyme engineering, enzyme mining, kinetic investigations, reaction engineering and recycling strategies will also be necessary to pave the way for enzymatic N–N bond formation beyond laboratory scale. As mentioned above, NNzymes currently still have their limitations and chemical synthesis remains an invaluable technique for the synthesis of N–N bond-containing compounds, however, there are certain limitations of chemical synthesis that could potentially be overcome with biocatalytic approaches.^25^ For example, the conventional chemical synthesis of conformationally constrained molecules such as Piz (**L-4**) requires the implementation of extensive protection and deprotection steps, which ultimately results in a reduction in overall yields.^237^ Furthermore, biologically active compounds often possess complex stereochemistry, necessitating meticulous control over the formation of chiral centers.^238^ Chemical synthesis frequently encounters difficulties in attaining the desired level of enantiomeric and diastereomeric purity.^239^ The presence of multiple functional groups can also result in unintended side reactions, whereas enzymes typically exhibit high specificity for the substrates of interest.^240^ In addition, chemical synthesis often requires the use of toxic or hazardous reagents (e.g., *n*-butyllithium and organoaluminum compounds) and solvents (e.g., chlorinated solvents such as carbon tetrachloride or 1,2-dichloroethane, ethers such as furan, hydrocarbons such as benzene or *o*-xylene),^241^ which inherently pose environmental and safety risks, while byproducts of biotechnological processes are frequently biodegradable.^242^ In this regard, genetic and metabolic engineering offers a distinct advantage over conventional chemical synthesis, as it can be employed to optimize pathways and obtain the final products with high efficiency.^243^ One illustrative example is the fermentative production of **L-4** in a genetically modified *Aureobasidium melanogenum* strain expressing KtzT.^80^ The formation of more than 10 g of **L-4** in 5 days in a 10-L reactor was demonstrated starting from 120 g of glucose. This method does not require organic solvents, intermediate purification steps or other chemical precursors. This example highlights that NNzymes represent one of many promising areas where enzymatic reactions can complement synthetic chemistry to access N–N bond-containing products in an efficient manner.

## 6 Concluding Remarks

The elucidation of the various pathways and reaction mechanisms leading to the formation of N–N bonds in NPs has become an area of emerging interest in the past decade. Thus, it is not surprising that a large number of NPs containing a variety of N–N bond-containing functional groups have been identified recently. To date, there are several functional groups that are known or predicted to be accessible through NNzymes. These groups are cyclic and linear hydrazines, nitroso- and diazo-compounds, triazenes and triazoles, which are also presented in this review in the context of the conventional methods for their synthesis. Nevertheless, our current understanding of how NNzymes facilitate N–N bond formation remains largely elusive, despite several of them having been conclusively linked to N–N bond-forming reactions through *in vitro* characterization, and structural as well as mechanistic information is available. Examples include the PZS, involved in the synthesis of cyclic hydrazines^79^ and the HDO/cupin-domain containing enzyme SznF/StzF, involved in the biosynthesis of nitrosamines.^215^ This is likely due to the diversity of enzymes and reaction mechanisms used in nature to form N–N bonds, and the difficulties related to the often low level of protein sequence identity and cofactor dependency. Moreover, N–N bond formation is often not directly enzyme-catalyzed, but occurs spontaneously, e.g. by radical recombination,^89^ after enzymatic formation of the reactive intermediates. Recently, it has been shown that in addition to enzymes that directly catalyze N–N bond formation, so-called hydrazine transferases catalyze the condensation of hydrazine and an aromatic polyketide intermediate to form a rare *N*-aminolactam pharmacophore,^179^ demonstrating that nature has evolved fascinating enzymes to enable such challenging chemistry. This finding further suggests that we do not yet understand all the mechanisms involved in biological N–N bond formation or the construction of complex N–N bond-containing NPs.

It is very likely that we have only seen a glimpse of the diversity of NNzymes to date. Thus, it is expected that the number of N–N bond-containing NPs and their corresponding biosynthetic NNzymes will continue to increase in the next few years. As more structural and mechanistic information on existing NNzymes becomes available, we expect that this knowledge will also support the functional elucidation of newly discovered NNzymes. Although currently minimally exploited, the growing information on PZS and hydrazine synthetases provides a solid basis for targeted protein engineering efforts, e.g. to increase substrate scope, catalytic efficiency or even catalytic promiscuity. Together, they could provide an exciting biocatalytic toolbox with largely untapped potential, e.g., for combining NNzymes in (chemo)enzymatic cascades with photo/photoredox, organo- or transition-metal catalysis. This would enable more challenging transformations for the synthesis of a wide variety of N–N bond-containing compounds used as pharmaceuticals, agrochemicals, coordination polymers, as well as organic and energy materials.

## Data Availability

The pdb file for the AlphaFold3 model of homodimeric KtzT-heme shown in Scheme 3 is available upon request from the corresponding author.

## Abbreviations

6-diazo-5-oxo-l-norleucine: (DON)
aspartate-nitrosuccinate: (ANS)
biosynthetic gene cluster: (BGC)
European Medicines Agency: (EMA)
heme-oxygenase–like diiron oxidase and oxygenase: (HDO)
The United States Food and Drug Administration’s Center for Drug Evaluation and Research: (FDA CDER)
methionyl-tRNA synthetase: (MetRS)
natural product: (NP)
*N*-hydroxylating monooxygenase: (NMO)
nitrogen–nitrogen: (N–N)
N–N bond-forming enzymes: (NNzymes)
nonribosomal peptide synthetase: (NRPS)
piperazate synthase: (PZS)
quantum mechanics/molecular mechanics: (QM/MM)
sequence similarity network: (SSN)
transition metal: (TM)
